# Engineered MATE multidrug transporters reveal two functionally distinct ion-coupling pathways in NorM from *Vibrio cholerae*

**DOI:** 10.1038/s42003-021-02081-6

**Published:** 2021-05-11

**Authors:** Sagar Raturi, Asha V. Nair, Keiko Shinoda, Himansha Singh, Boyan Bai, Satoshi Murakami, Hideaki Fujitani, Hendrik W. van Veen

**Affiliations:** 1 Department of Pharmacology, University of Cambridge, Cambridge, UK; 2 Microbial Membrane Transport Engineering, Biotechnology Research Center, The University of Tokyo, Bunkyo-ku, Tokyo Japan; 3 Department of Life Science and Technology, Tokyo Institute of Technology, Nagatsuta, Midori-ku, Yokohama Japan; 4 Laboratories for Systems Biology and Medicine, Research Center for Advanced Science and Technology, The University of Tokyo, Meguro-ku, Tokyo Japan; 5 Present Address: University College Dublin Clinical Research Centre, St. Vincent’s University Hospital, Dublin, Ireland

**Keywords:** Enzyme mechanisms, Antimicrobial resistance

## Abstract

Multidrug and toxic compound extrusion (MATE) transport proteins confer multidrug resistance on pathogenic microorganisms and affect pharmacokinetics in mammals. Our understanding of how MATE transporters work, has mostly relied on protein structures and MD simulations. However, the energetics of drug transport has not been studied in detail. Many MATE transporters utilise the electrochemical H^+^ or Na^+^ gradient to drive substrate efflux, but NorM-VC from *Vibrio cholerae* can utilise both forms of metabolic energy. To dissect the localisation and organisation of H^+^ and Na^+^ translocation pathways in NorM-VC we engineered chimaeric proteins in which the N-lobe of H^+^-coupled NorM-PS from *Pseudomonas stutzeri* is fused to the C-lobe of NorM-VC, and vice versa. Our findings in drug binding and transport experiments with chimaeric, mutant and wildtype transporters highlight the versatile nature of energy coupling in NorM-VC, which enables adaptation to fluctuating salinity levels in the natural habitat of *V. cholerae*.

## Abstract (web summary)

Raturi and Nair et al. describe functional ion pathways within the MATE transporter NorM from *Vibrio cholerae* (NorM-VC). By creating chimeric proteins and mutants of NorM-VC and NorM-PS, they dissect the localisation of ion-coupling events and roles of conserved catalytic carboxylates in H^+^ binding and Na^+^ coordination, with findings useful to the field of transporters and drug resistance pumps.

## Introduction

**Fig. 1:**
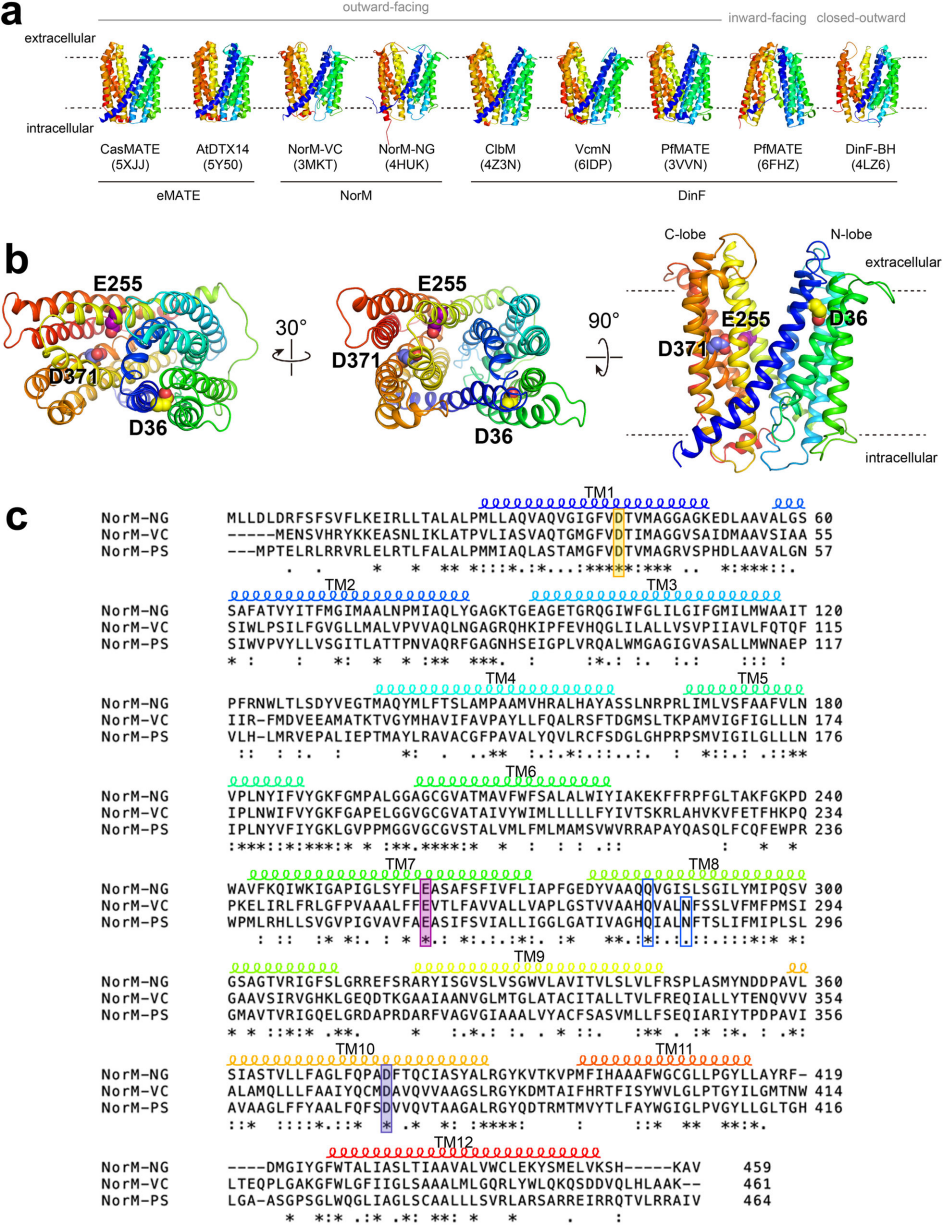
Structural features of MATE transporters. **a** Overview of MATE subfamilies showing the V-shaped outward-facing structures of plant eMATE transporters from *Camelina sativa* (CasMATE, PDB-ID: 5XJJ)^9^ and *Arabidopsis thaliana* (AtDTX14, PDB-ID: 5Y50)^10^, NorM transporters from *Vibrio cholerae* (NorM-VC, PDB-ID: 3MKT)^7^ and *Neisseria gonorrhoeae* (NorM-NG, PDB-ID: 4HUK)^8,13^ and DinF transporters from *Escherichia coli* (ClbM, PDB-ID: 4Z3N)^60^, *V. cholerae* (VcmN, PDB-ID: 6IDP)^14^ and *Pyrococcus furiosus* (PfMATE, PDB-ID: 3VVN)^11^. In these structures the N- and C-terminal halves, referred to N- and C-lobes, contact each other at the intracellular side. Also shown are the inward-facing conformation of PfMATE (PDB-ID: 6FHZ)^16^ and closed-outward conformation of DinF-BH from *Bacillus halodurans* (PDB-ID: 4LZ6)^12^. Transmembrane helices (TMHs) are shown in *rainbow colours* with TMH1-6 in blue–green and TMH7-12 in yellow–orange–red. Dotted lines depict the approximate membrane boundaries. **b** Structure of NorM-VC (PDB-ID: 3MKT)^7^ in three different orientations, showing the locations of catalytic carboxylates D36 in the N-lobe and E255 and D371 in the C-lobe as spheres. **c** Alignment of amino acid sequences for NorM-VC (AAF94694.1) from *Vibrio cholerae* serotype O1 (ATCC 39315), NorM-PS (EHY79494.1) from *Pseudomonas stutzeri* strain ZoBell (ATCC 14405) and NorM-NG (WP_003687823.1) from *Neisseria gonorrhoeae*. The alignment indicates the location of the transmembrane helices and shows that D36, E255 and D371 in NorM-VC are conserved as D38, E257 and D373 in NorM-PS, and D41, E261 and D377 in NorM-NG. The conservation of residues Q278 and N282 in NorM-VC (see Fig. 9) is indicated in the open blue boxes. The protein structures were prepared in Pymol v2.4.0; the sequence alignment was generated in Clustal Omega v1.2.4.

The members of the multidrug and toxic compound extrusion (MATE) family are widely distributed in bacteria, archaea, and eukarya^1^. The MATE family belongs to the multidrug/oligosaccharidyl-lipid/polysaccharide (MOP) transporter superfamily which, based on sequence similarity, is further classified in prokaryotic NorM and DinF subfamilies and the eukaryotic MATE (eMATE) subfamily^2,3^ (Fig. 1a). The bacterial MATE proteins transport amphiphilic cationic drugs, such as norfloxacin and ethidium, from the cellular interior. Plant MATE transporters have physiological roles in herbicide resistance, sequestration of plant-derived organic compounds in vacuoles, leaf senescence, aluminium tolerance, iron homoeostasis, and synthesis of auxins^4^. In mammals, MATE transporters are localised in the proximal convoluted tubule and proximal straight tubule in the kidney, as well as the canalicular membrane in hepatocytes, where they mediate transport of organic cations in the final steps of drug elimination from the body^3,5^. Owing to their involvement in a wide range of physiological processes, MATE transporters are attractive pharmaceutical targets.

The structures of MATE transporters show that these proteins contain 12 transmembrane helices (TMs) that are organised in two 6 TM domains, referred to as N-lobe and C-lobe (Fig. 1a, b). These helices are arranged differently from those in major facilitator superfamily (MFS) transporters^6,7^, supporting earlier conclusions that MATE and MFS proteins should be regarded as separate protein families^2^. Over the past decades, the evolutionary persistence of alternating access mechanisms has been shown for a variety of membrane transporters. Via sequential conformational changes, the transporter alternates between two major conformations, one of which exposes a central substrate-binding pocket to the cellular interior, and the other exposes this pocket to the exterior of the cell. For MATE transporters, the structural details and catalytic reactions underlying the necessary conformational changes are still only partly understood. In part, this is because all, but two crystal structures of MATE transporters have been reported in the outward-facing conformation (Fig. 1a).

Transporters in the MATE family were originally described to mediate drug/ion antiport in a Na^+^ or H^+^-coupled fashion. X-ray crystallographic studies suggest that the NorM subfamily is dependent on Na^+^ coupling in the C-lobe^7,8^, the eMATE subfamily requires H^+^ coupling in the C-lobe^9,10^, and the DinF subfamily utilises H^+^ coupling in the N-lobe^11–15^. Further structural investigations on two members of the DinF family suggest the presence of a Na^+^-binding site in their N-lobes, but this Na^+^ dependency was not demonstrated in drug transport measurements or biochemical assays^16–18^. In fact, as the progress in the MATE transporter field has mostly relied on protein structural techniques^19^, the energetics of MATE transporters has not been studied systematically with well-established methods in proteoliposomes and intact cells, developed over decades of transporter research. Therefore, the ion/drug stoichiometries and dependencies of the antiport reactions on chemical ion gradients and the membrane potential are mostly unknown. Using these methods in our previous study, we identified the ability of NorM from *Vibrio cholerae* (NorM-VC) to utilise both the H^+^ and Na^+^ gradient in its drug-efflux activity^20^. Follow-up biophysical measurements on NorM-VC based on double-electron electron resonance (DEER) spectroscopy confirmed the existence of Na^+^- and H^+^-bound intermediates in NorM-VC^21^. Here, we study the ion-translocation pathways in NorM-VC and their bioenergetic contributions to drug efflux in transport measurements, drug binding assays and molecular dynamics (MD) simulations. In contrast to NorM-VC, the NorM orthologue in *Pseudomonas stutzeri* (NorM-PS) (Fig. 1c) was reported to be solely dependent on H^+^ coupling^22,23^. We prepared NorM-VC/NorM-PS chimaeras and mutant proteins that enabled us to dissect the localisation of ion-coupling events and roles of conserved catalytic carboxylates in H^+^ binding and Na^+^ coordination.

## Results

### H^+^ and Na^+^ dependence in NorM-VC-mediated ethidium efflux

**Fig. 2:**
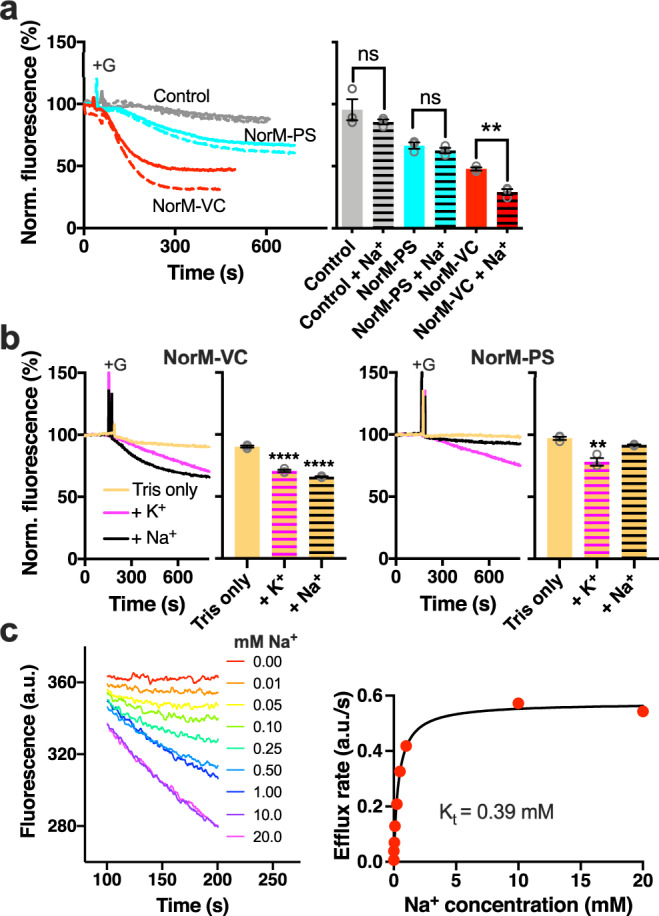
Comparison of Na^+^ dependency of NorM-VC and NorM-PS. **a** Upon the addition of 20 mM glucose (+G), cells expressing NorM-VC (red traces) or NorM-PS (cyan traces) show significant ethidium efflux over time compared to non-expressing control cells (grey traces). The presence of 1 mM Na^+^ in buffer pH 7.0 (dotted traces) stimulates efflux activity by NorM-VC but not by NorM-PS. The histogram on the right shows mean levels of ethidium fluorescence at 400 s in repeat experiments. Open bars, -Na^+^. Horizontally-striped bars, +Na^+^. **b** In a K^+^-free Tris buffer, ethidium efflux by NorM-VC in glucose-metabolising cells (yellow trace) is stimulated by the addition of 10 mM Na^+^ (black trace) or K^+^ (pink trace), indicating that the ΔpNa and ΔpH can each be a driving force for transport. In contrast, ethidium efflux by NorM-PS is only stimulated by K^+^ but not by Na^+^. Histograms show mean levels of ethidium fluorescence at 600 s in repeat experiments. Open yellow bars, MATE transporter-expressing cells in Tris buffer. Black or pink horizontally-striped bars, +Na^+^ or +K^+^, respectively. **c** Active ethidium efflux by NorM-VC as a function of the Na^+^ concentration (rainbow coloured) in buffer pH 7.0. A plot of the efflux rates versus Na^+^ concentrations was fitted to a hyperbola. Data represent observations in three experiments with independently prepared batches of cells. Values are expressed as mean ± s.e.m. (two-way analysis of variance; **P < 0.01; ****P < 0.0001). Asterisks above square brackets refer to comparisons with the no-Na^+^ condition (**a**), whereas asterisks directly above bars refer to comparisons with Tris buffer only (**b**); ns indicates ‘not significant’.

Our investigations focussed on NorM proteins expressed in *Lactococcus lactis* Δ*lmrA* Δ*lmrCD*. This organism is ideal for this purpose due the reduced expression of endogenous multidrug transporters and the ease of manipulation of bioenergetic parameters in substrate transport measurements (see ‘Bioenergetic considerations’ in ‘Methods’). As ethidium shows an enhancement of fluorescence emission upon binding to nucleic acids in the cellular interior, the efflux of ethidium is associated with a fluorescence decrease. Following the addition of glucose to ethidium-loaded cells expressing NorM-VC, the generation of ATP and proton motive force (Δp, interior negative and alkaline) activate ethidium efflux (Fig. 2a). This efflux was significantly enhanced when 1 mM Na^+^ was added to the buffer. To further study these transport reactions, cells were suspended in Tris buffer. The lack of K^+^ in this buffer disables the membrane potential (Δψ, interior negative)-dependent K^+^ uptake in the cells that allows the controlled dissipation of the Δψ and establishment of a transmembrane chemical proton gradient (ΔpH, interior alkaline) during proton export by the plasma membrane F_0_F_1_ H^+^-ATPase^24^. The restoration of robust ethidium efflux following the addition of 10 mM K^+^ to the buffer points to the importance of the ΔpH as a driving force for ethidium efflux (Fig. 2b). The establishment of a sodium motive force (Δp_Na_, interior negative and low) in these cells, facilitated by the addition of 10 mM Na^+^ instead of K^+^, also significantly stimulated ethidium efflux by NorM-VC. Determination of initial ethidium efflux rates as a function of the Na^+^ concentration yielded an apparent affinity constant K_t_ of 0.39 ± 0.06 mM Na^+^ (Fig. 2c), which is comparable to previously reported data^20^. These results suggest that NorM-VC can use both Na^+^ and H^+^ as coupling ions. Strikingly, when these experiments were repeated with lactococcal cells expressing the NorM-VC homologue NorM-PS from *P. stutzeri*^22,23^, the ethidium efflux reaction was stimulated by K^+^ but not by Na^+^ (Fig. 2a, b). Therefore, the mechanisms of energy coupling to ethidium efflux in NorM-VC and NorM-PS are different.

### Effect of the H^+^ and Na^+^ availability in ion coupling in NorM-VC

**Fig. 3:**
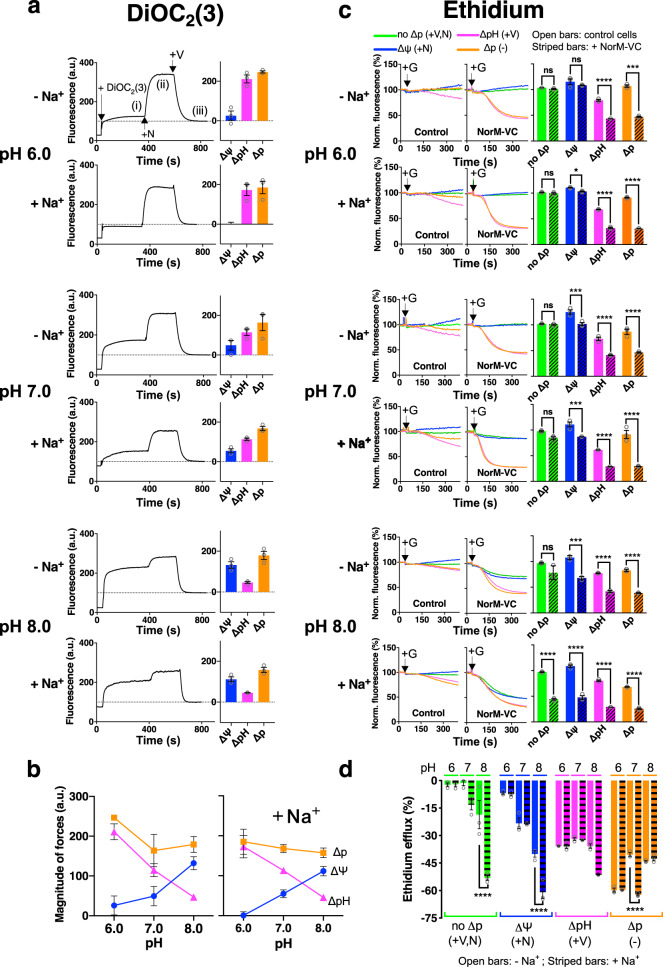
Energetics of H^+^- and Na^+^-coupled ethidium efflux in NorM-VC. **a** The composition of the ∆p in cells suspended in buffer pH 6.0, 7.0, and 8.0 was assessed using the fluorescent membrane potential probe DiOC_2_(3). When added to control cells, the probe reported three plateau levels in fluorescence emission (see top panel). Level (i) reflects probe accumulation in the absence of ionophores due to the ∆ψ (interior negative) in glucose-energised cells in which ∆p = ∆ψ − Z∆pH. Level (ii) reports further probe accumulation following the addition of 0.5 µM nigericin (+N), which causes the interconversion of the ∆pH into extra ∆ψ and provides a measure for the ∆p (=∆ψ + extra ∆ψ). Level (iii) follows the addition of 0.1 µM valinomycin (+V), which causes the dissipation of the ∆ψ and decrease of the DiOC_2_(3) fluorescence to baseline level. The histograms on the *right* represent ∆ψ = level (i) – level (iii) (blue bars), ∆pH = level (ii) − level (i) (pink bars) and ∆p = level (ii) – level (iii) (orange bars). The additions of the probe (+DiOC_2_(3)), nigericin (+N) and valinomycin (+V) are labelled in the top panel. **b** Magnitudes and compositions of the ∆p in (**a**) as a function of buffer pH. **c** Effect of the buffer pH 6.0, 7.0 and 8.0 on ethidium efflux in NorM-VC expressing cells or non-expressing control cells in absence of ionophores (∆p) (*orange traces*) or presence of nigericin (∆ψ only) (blue traces), valinomycin (∆pH only) (pink traces), or both ionophores (no ∆p) (green traces). The ionophores were added 3 min prior to the addition of the glucose (+G). Measurements were performed in the absence or presence of 1 mM Na^+^ as indicated on the left. Histograms on the right show levels of ethidium fluorescence at 300 s in repeat experiments. Open bars, non-expressing control cells. Diagonally-striped bars, NorM-VC-expressing cells. **d** Ethidium efflux activity as a function of buffer pH and driving force, in absence of Na^+^ (open bars) or presence of Na^+^ (horizontally-striped bars). For calculation of bar heights, ethidium fluorescence levels at 300 s in (**c**) for cells without NorM-VC expression were subtracted from corresponding levels for cells with NorM-VC expression. Data represent observations in three experiments (*n* = 3) with independently prepared batches of cells. Values are expressed as mean ± s.e.m. (two-way analysis of variance; *P < 0.05; ***P < 0.001; ****P < 0.0001). The asterisks above or below square brackets refer to comparisons with the non-expressing control in (**c**) or no-Na^+^ condition in (**d**); ns indicates ‘not significant’.

To study energy coupling by NorM-VC in more detail, ethidium transport in cells was measured at different concentrations of H^+^ and Na^+^ in the external buffer under conditions where the magnitude and composition of the ∆p was manipulated with the ionophores nigericin and/or valinomycin. The effect of the ionophores was first tested in measurements of the ∆ψ using DiOC_2_(3) (Fig. 3a, b). This lipophilic cationic probe accumulates in the interior of cells at the negative side of the polarised plasma membrane, where the increased probe concentration is associated with fluorescence intensity enhancement. Following the sequential additions of glucose, nigericin and valinomycin to the cells, three levels of DiOC_2_(3) fluorescence are observed (indicated in the top panel of Fig. 3a). Level (i) reflects the ∆ψ (interior negative) in glucose-metabolising cells in the absence of the ionophores. Level (ii) was reached following the addition of 0.5 µM nigericin. Nigericin mediates electroneutral (Δψ-independent) H^+^/K^+^ exchange across the plasma membrane, which leads to the interconversion of ∆pH into extra ∆ψ (interior negative)^25^, transforming ∆p = ∆ψ − Z∆pH into ∆p = ∆ψ + extra ∆ψ. Finally, Level (iii) was reached following the addition of 0.1 µM valinomycin, which mediates electrogenic (Δψ-dependent) K^+^ uniport across the plasma membrane and causes the dissipation of the ∆ψ and reduction of the DiOC_2_(3) fluorescence to baseline level. These measurements demonstrate that the ∆p is relatively constant at an external pH of 6.0, 7.0 and 8.0 (Fig. 3b). Due to homeostasis of the internal pH in the cells (pH_in_ is constant at approx. 7.8–8.0)^24^, the contribution of the ∆pH to the ∆p is highest at pH 6.0 whereas the contribution of the ∆ψ is highest at pH 8.0. The addition of 1 mM Na^+^ did not cause significant shifts in the magnitude and composition of the ∆p at the different pH values (Fig. 3b).

Having established the effect of ionophores on the ∆ψ, ∆pH and ∆p in our lactococcal cells, the ionophores were used in ethidium efflux experiments with the cells suspended in KPi buffer pH 6.0, 7.0 and 8.0 in the absence or presence of 1 mM Na^+^ (Fig. 3c). At pH 6.0, the active efflux of ethidium by NorM-VC in the presence of nigericin (∆ψ only) was similar to the control where both ionophores were added simultaneously (no ∆p). This is due to the low internal pH that is inhibitory for NorM-VC activity (pH_in_ equals the pH of buffer in which cells are suspended). However, in the presence of valinomycin (∆pH only) or absence of ionophores (∆p), NorM-VC was transport-active; this activity was not affected by the inclusion of 1 mM Na^+^ in the external buffer. These data point to the dominance of proton coupling and lack of Na^+^ coupling to ethidium efflux at pH 6.0. In further descriptions of ethidium transport experiments, the ionophore additions will not be repeated. Instead, we will refer to the electrochemical forces driving the efflux (∆p, ∆pH, ∆ψ or none) that are present in the cells. At pH 7.0 and pH 8.0, where the H^+^ concentration is 10-fold and 100-fold lower compared to pH 6.0, ethidium efflux by NorM-VC was dependent on both the ∆ψ and ∆pH, consistent with electrogenic antiport of ethidium^+^ and two or more protons (Fig. 3c). The imposition of an inwardly-directed chemical Na^+^ gradient (∆pNa, interior low) significantly stimulated ethidium efflux in NorM-VC-expressing cells relative to non-expressing control cells in the presence of the ∆p at pH 7.0 and ∆pH and ∆ψ at pH 8.0 (Fig. 3d). The ∆pNa also significantly stimulated NorM-VC-dependent ethidium efflux for the no-∆p control at pH 8.0. Therefore, no further stimulation of ∆pNa-dependent efflux activity at this pH was obtained by the additional imposition of the ∆p (Fig. 3d). Two conclusions can be drawn from these experiments. First, similar to H^+^-coupled ethidium efflux by NorM-VC, Na^+^-coupled ethidium efflux is based on an electrogenic transport reaction. Second, Na^+^ coupling in NorM-VC is more pronounced for cells in buffer pH 8.0 than 6.0 or 7.0, indicating that the ability of NorM-VC to couple to the ∆p and ∆p_Na_ is affected by the relative availability of Na^+^ and H^+^ in the external environment.

### Biochemical studies on Na^+^ and H^+^ binding in NorM-VC

**Fig. 4:**
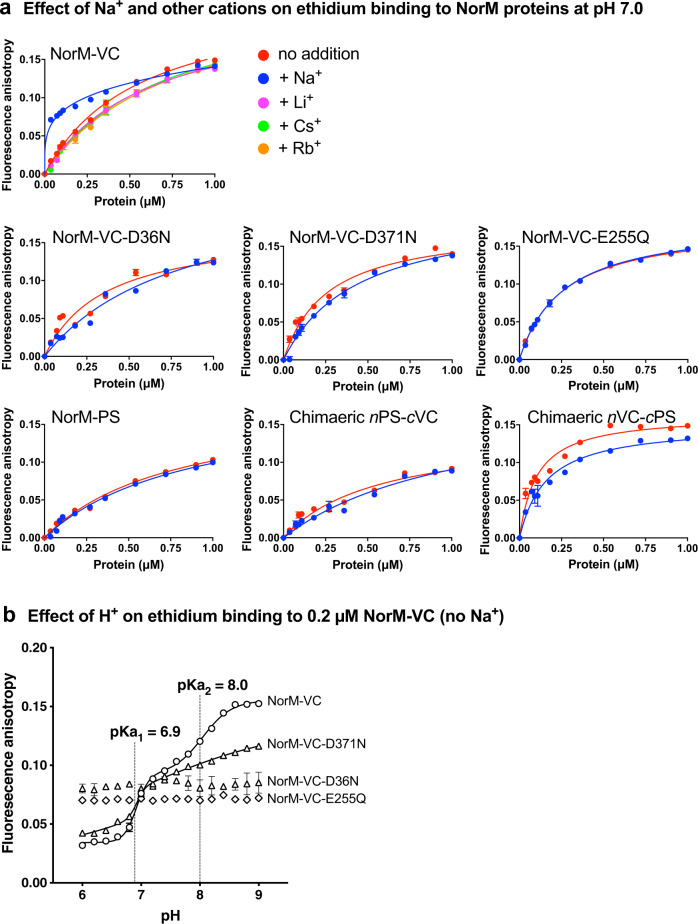
Equilibrium ethidium binding to NorM proteins. **a** Purified NorM proteins in detergent solution were added stepwise to buffer pH 7.0 containing 2 μM ethidium in the absence of Na^+^ (*red symbols*) or presence of 1 mM Na^+^ (blue symbols) or Li^+^, Cs^+^, or Rb^+^ (purple, green and orange symbols, respectively), after which the fluorescence anisotropy was measured. Na^+^ stimulates high-affinity binding of ethidium to wildtype NorM-VC but not to the NorM-VC mutants D36N, D371N and E255Q, or NorM-PS or chimaeric proteins. **b** Ethidium binding to NorM-VC proteins was measured as a function of buffer pH at 0.2 µM purified NorM-VC and 2 µM ethidium. Ethidium binding to wildtype protein exhibits a double sigmoidal increase as the pH is raised, from which two midpoint pKa values of 6.9 and 8.0 were derived by non-linear curve fitting. Data represent observations in three experiments with independently prepared batches of purified proteins. Values are expressed as mean ± s.e.m. Some error bars are hidden behind the data point symbols.

The interaction of NorM-VC with Na^+^ and H^+^ was further investigated in a biochemical assay in which ethidium binding was used as a reporter (Fig. 4a). Samples of freshly purified proteins in detergent solution were added in a stepwise manner to a KPi buffer (pH 7.0) containing a fixed 2 µM concentration of ethidium. At each addition of protein, the fluorescence anisotropy was recorded for about 2 min until a constant value was reached. In the presence of 1 mM Na^+^, the ethidium binding data were biphasic, with high-affinity binding superimposed on low-affinity binding. Non-linear fitting of the data to a two-sites specific-binding model revealed an apparent K_d_ below 40 nM protein for high-affinity binding and an apparent K_d_ above 0.2 µM protein for low-affinity binding (Fig. 4a). In the absence of Na^+^, the ethidium binding data reflect hyperbolic low-affinity binding only, due to the loss of the high-affinity ethidium binding. These results indicate that the interaction of Na^+^ with NorM-VC stabilises a conformation that binds ethidium with high affinity. In a dynamic equilibrium, this conformation coexists with Na^+^-free conformations exhibiting low-affinity ethidium binding. The results are consistent with the enhanced UV cross-linking of azido-ethidium to NorM-VC in the presence of Na^+^^20^.

NorM-VC contains conserved carboxyl residues, D36 at the external face of the N-lobe and E255 and D371 at the centre of the C-lobe (Fig. 1b), that might catalyse proton binding and/or, when deprotonated, contribute to Na^+^ coordination. When the ethidium binding assays were repeated with mutants D36N, E255Q, and D371N, containing single carboxyl-to-amide substitutions that mimic the protonated carboxylic acid side chain, the Na^+^-dependent high-affinity ethidium binding was lost (Fig. 4a). Therefore, the N- and C-lobe of NorM-VC might contain a Na^+^ binding pocket. Indeed, crystal structures of NorM-VC (PDB-ID: 3MKT and 3MKU) have the Na^+^ analogues Rb^+^ or Cs^+^ bound near E255 and D371. For NorM from *Neisseria gonorrhoeae* (PDB-ID: 4HUL) a Cs^+^ ion is bound at the equivalent position. However, in our assays with wildtype NorM, ethidium binding was low-affinity in the presence of 1 mM Cs^+^, Rb^+^ or Li^+^ (Fig. 4a) suggesting that, although these Na^+^ analogues might mimic a bound Na^+^ ion in the crystal structures, they do not mimic Na^+^ binding at the functional level. This might relate to the differences in atomic radii and coordination chemistry of these ions.

In biochemical studies on H^+^ interactions in NorM-VC, the binding of ethidium to wildtype protein increased dramatically and in a double sigmoidal fashion when the buffer pH was increased from pH 6 to 9 (Fig. 4b). The two midpoints at pH 6.9 and 8.0 suggest apparent pKa values of proton binding to NorM-VC. Among the D36N, E255Q and D371N mutants, only D371N retained ethidium binding, but with a much lower rise around the second midpoint. Given that the pKa values of carboxylates near the external membrane surface will be lower than those near the internal surface to allow protonation and deprotonation in response to a ∆pH (interior alkaline), it is likely that the first midpoint represents the pKa of D36 while the second midpoint represents the sum of pKa values of E255 and D371. These data reinforce previous observations that proton release is required for ethidium binding to NorM-VC^20^. When taken together, the results from these biochemical studies highlight the impact of Na^+^ and H^+^ interactions in NorM-VC on ethidium binding to the transporter.

### Role of catalytic carboxylates in energy coupling in NorM-VC

**Fig. 5:**
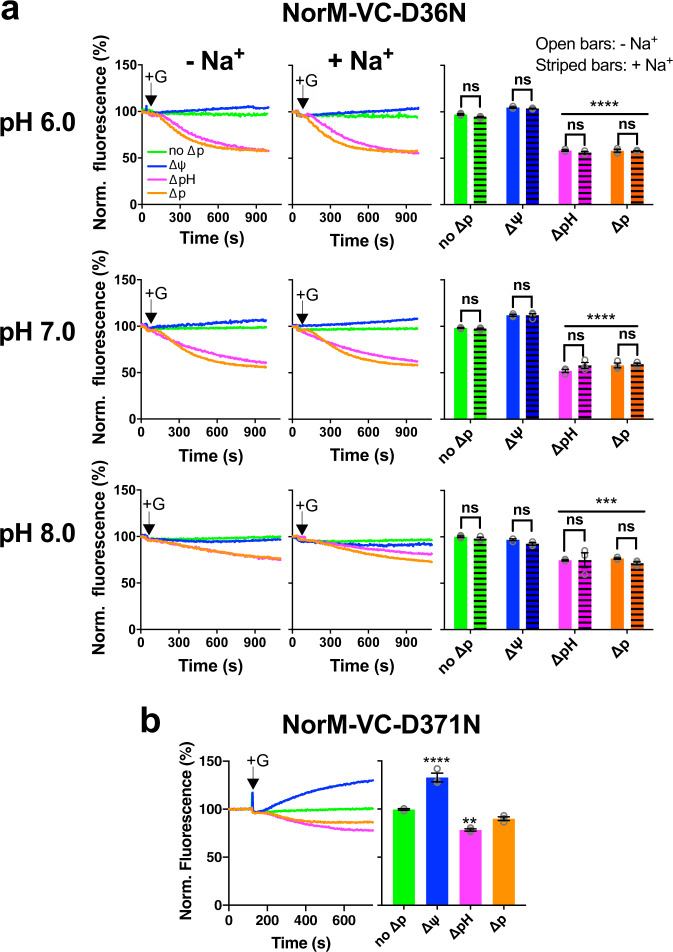
Role of catalytic carboxylates D36 and D371 in ethidium efflux by NorM-VC. **a** Effect of the buffer pH 6.0, 7.0 and 8.0 on ethidium transport by the D36N mutant in the absence of the ∆p (no ∆p) (green traces) or the presence of ∆ψ (blue traces), ∆pH (pink traces) or ∆p (orange traces). Glucose (+G) and ionophores were added to the cells as described in Fig. 3c. The histograms show levels of ethidium fluorescence at 900 s in repeat experiments in the absence of Na^+^ (open bars) or in the presence of 1 mM Na^+^ (horizontally-striped bars). The data show that ∆ψ is not a driving force for efflux by the D36N mutant, pointing to electroneutral ethidium^+^/H^+^ antiport. **b** Measurements of ethidium efflux by the D371N mutant also indicate electroneutral ethidium^+^/H^+^ antiport. Data represent observations in three experiments (*n* = 3) with independently prepared batches of cells. Values indicate mean ± s.e.m. (two-way analysis of variance; **P < 0.01; ***P < 0.001; ****P < 0.0001). Asterisks above square brackets refer to comparisons with the no-Na^+^ condition, whereas asterisks directly above bars or groups of bars (indicated by horizontal line) refer to comparisons with the no-∆p control; ns indicates ‘not significant’.

The contribution of catalytic carboxylates to energy coupling in NorM-VC was studied in more detail. The D36N mutants exhibited significant ethidium efflux activity in the presence of the ∆pH or ∆p (Fig. 5a) relative to the no-∆p condition. However, different from wildtype NorM-VC (Fig. 3c, d), no efflux activity was seen in the presence of the ∆ψ only. Furthermore, the Na^+^-dependent stimulation of ethidium efflux by wildtype NorM-VC was lost for the D36N mutant (Fig. 5a). Clearly, the mutant lacks the input of H^+^ or Na^+^ that usually enters the reaction via D36 at the external side of the transporter and, as a consequence, mediates electroneutral ethidium^+^/H^+^ antiport rather than the electrogenic ethidium^+^/(H^+^, Na^+^) antiport observed for wildtype NorM-VC. Regarding the E255-D371 region, a previous study established the complete inhibition of ethidium transport in the E255Q mutant as well as electroneutral ethidium/Na^+^ antiport by the D371N mutant^20^. We repeated these measurements for NorM-VC-D371N in the absence of Na^+^ and obtained evidence for electroneutral ethidium/H^+^ antiport (Fig. 5b). These findings highlight the importance of the D36 and E255-D371 regions for the coupling of NorM-VC to the electrochemical Na^+^ and H^+^ gradients.

### Ion coupling in NorM-PS differs from NorM-VC

**Fig. 6:**
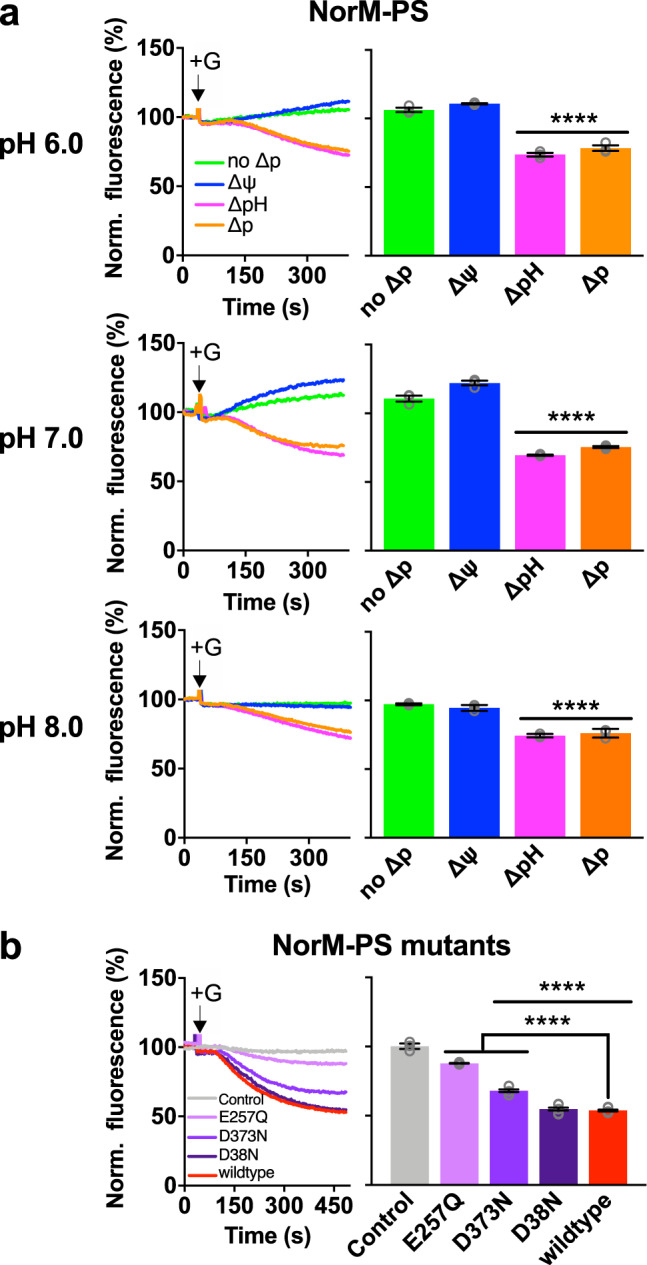
Transport studies on NorM-PS proteins. **a** Effect of buffer pH 6.0, 7.0 or 8.0 on ethidium efflux by wildtype NorM-PS-expressing cells in the absence of the ∆p (no ∆p) (green traces) or the presence of ∆ψ (blue traces), ∆pH (pink traces), or ∆p (orange traces). Glucose (+G) and ionophores were added to the cells as described in Fig. 3c. Histograms show levels of ethidium fluorescence at 350 s in repeat experiments. **b** Ethidium efflux in glucose-metabolising cells expressing NorM-PS mutants containing carboxyl-to-amide replacements (purple traces and bars). The activity is compared with non-expressing control cells (grey trace and bar) and cells expressing wildtype NorM-PS (red trace and bar). Histograms show levels of ethidium fluorescence at 450 s in repeat experiments. Data represent observations in three experiments (*n* = 3) with independently prepared batches of cells. Values are expressed as mean ± s.e.m. (one-way analysis of variance; ****P < 0.0001). Asterisks directly above groups of bars (indicated by horizontal line) refer to comparisons with the no-∆p control (**a**) or non-expressing control (**b**). The square bracket with asterisks in **b** refers to the comparison with wildtype NorM-PS.

In similar experiments for NorM-PS, this transporter showed a significant ethidium efflux activity in the presence of the ∆pH or ∆p (Fig. 6a) but no efflux activity with the ∆ψ only. The energetics of ethidium efflux by NorM-PS is, therefore, comparable to NorM-VC-D36N and consistent with electroneutral ethidium^+^/H^+^ antiport. Regarding the conserved catalytic carboxylates (Fig. 1c), the D38N mutation in the N-lobe had no noticeable effects on ∆p-dependent ethidium efflux activity relative to wildtype NorM-PS (Fig. 6b), whereas the E257Q and D373N mutations in the C-lobe significantly inhibited ethidium efflux with residual activity for the D373N mutant relative to the non-expressing control (Fig. 6b). Finally, neither ethidium efflux (Fig. 2a, b) nor ethidium binding (Fig. 4a) were stimulated in the presence of 1 mM Na^+^ suggesting that wildtype NorM-PS is unable to use Na^+^ in its transport mechanism. When taken together, these results indicate that, unlike D36 in NorM-VC, D38 in NorM-PS is catalytically silent in our measurements.

**Fig. 7:**
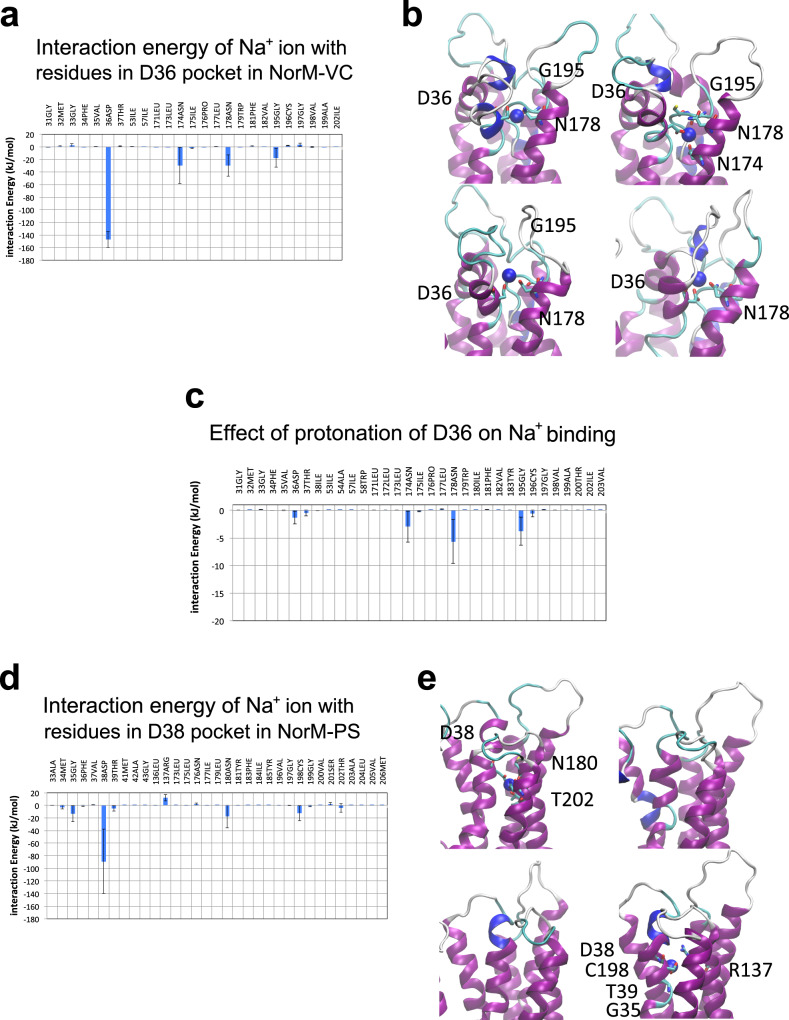
MD simulations of the dissociation of pre-bound Na^+^ from the D36/D38 pockets in outward-facing NorM-VC/NorM-PS. **a** Interaction energies between bound Na^+^ and residues in the D36 pocket within 0.8 nm distance of the Na^+^ were averaged over four MD simulations from 90 to 100 ns. **b** Close-up view of the D36 pocket at 100 ns for each of the four runs. The interacting residues are indicated in stick representation. **c** Na^+^ dissociation from the D36 pocket is enhanced by protonation of D36. These simulations show that the pre-bound Na^+^ ion (blue sphere) was retained in all four simulations for NorM-VC and was released from the pocket when the anionic charge in the D36 side chain was neutralised by protonation of the carboxylate. **d**, **e** MD simulations similar to those in (**a**, **b**) for the D38 pocket in NorM-PS. In two out of four simulations with NorM-PS, Na^+^ was not retained but diffused away from the D38 pocket within 100 ns. These simulations suggest that the D36 pocket binds Na^+^ more easily than the D38 pocket. Values are expressed as mean ± s.e.m.

The ability of Na^+^ to interact with D36 in NorM-VC and D38 in NorM-PS was also compared in molecular dynamics (MD) simulations in two complementary approaches. First, in a Na^+^ dissociation study, four independent MD simulations were performed with outward-facing NorM-VC with pre-bound Na^+^ in a binding pocket in the N-lobe that is formed by deprotonated D36, N174, N178 and G195 (Fig. 7a, b). When the MD simulations were started, the pre-bound Na^+^ ion was retained in all four simulations for NorM-VC (Fig. 7b) and was released from the pocket upon protonation of D36 (Fig. 7c). In contrast, in two of four simulations with deprotonated D38 in NorM-PS, Na^+^ was not retained but diffused away from the D38 pocket within 100 ns (Fig. 7d, e). Based on contributions to Na^+^ binding of residues within 0.8 nm of the ion, the calculated interaction energy for NorM-VC in the last 10 ns was significantly larger than for NorM-PS (−215 ± 30 kJ/mol versus −129 ± 85 kJ/mol, respectively). These simulations suggest that the D36 pocket in NorM-VC retains bound Na^+^ more easily than the D38 pocket in NorM-PS. Second, in a Na^+^ association study, the interaction of Na^+^ with the D36 and D38 pockets was compared by simulating the movement of the free ion into the empty pockets over a time span of 500 ns. When the D36 pocket in NorM-VC was exposed to Na^+^ at the start of the MD simulation, the ion associated with the D36 pocket in three out of four simulations with NorM-VC (Fig. S1). However, no binding of Na^+^ in the D38 pocket was observed in the four simulations with NorM-PS (Fig. S1). Evidently, despite the extensive sequence similarity between NorM-VC and NorM-PS (Fig. 1c), these proteins differ in their Na^+^ binding properties. It is noteworthy that when the molecular electrical potential surfaces at the external side of outward-facing NorM-VC and NorM-PS are compared, NorM-VC is more electronegative due to the presence of acidic surface residues. These are replaced by basic and hydrophobic residues in NorM-PS (Fig. S2). As a result of this difference, NorM-VC will have a stronger electrostatic attraction for Na^+^ ions in the external buffer.

### Fusions of N- and C-lobes of NorM-VC and NorM-PS give rise to transport-active chimaeric transporters

**Fig. 8:**
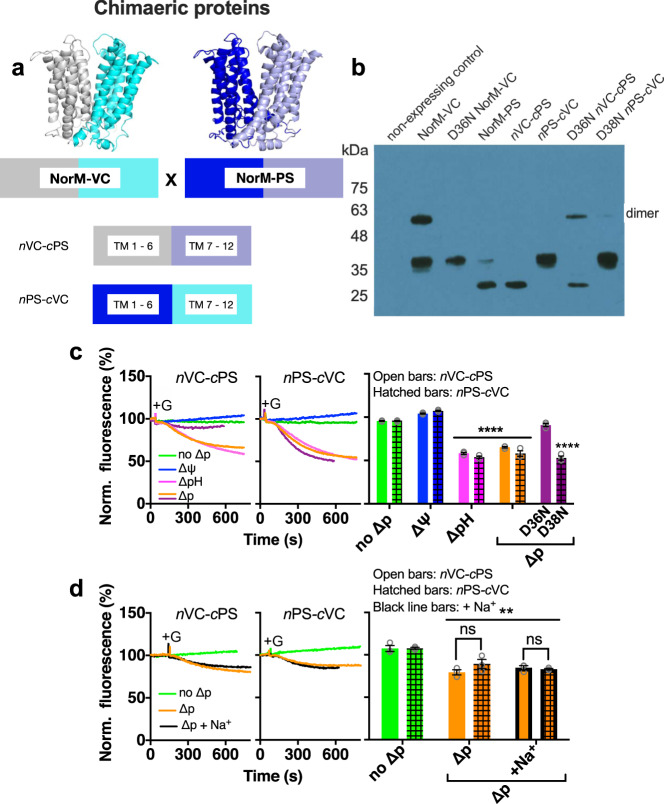
Ethidium transport by chimaeric NorM-VC/NorM-PS proteins. **a** Construction of chimaeric proteins *n*VC-*c*PS and *n*PS-*c*VC from NorM-VC and NorM-PS (see also main text). **b** Immunoblot probed with anti-His-tag antibody shows comparable expression of NorM-VC, NorM-PS and chimaeric proteins in plasma membrane vesicles (2 µg total membrane protein/lane). The uncropped blot is shown in Fig. S3. **c** Ethidium efflux by *n*VC-*c*PS (open bars) and *n*PS-*c*VC (hatched bars) at pH 7.0 in the absence of the ∆p (no ∆p) (green traces) or the presence of ∆ψ (blue traces), ∆pH (pink traces), or ∆p (orange traces). Glucose (+G) and ionophores were added to cells as described in Fig. 3c. Activity of D36N and D38N mutants is indicated in purple. The histograms on the right show levels of ethidium fluorescence at 500 s in repeat experiments for *n*VC-*c*PS (open bars) or *n*PS-*c*VC (hatched bars). The data show that both chimaeric proteins mediate electroneutral H^+^/ethidium^+^ antiport. **d** Ethidium efflux by the chimaeric proteins is not significantly stimulated by 1 mM Na^+^ in buffer pH 7.4 (black traces and horizontally-striped bars), where Na^+^ has 25,000-fold excess over H^+^. Data represent observations in three experiments (*n* = 3) with independently prepared batches of cells. Bar heights indicate mean ± s.e.m. (two-way analysis of variance; **P < 0.01; ****P < 0.0001). Asterisks directly above bars or groups of bars (indicated by horizontal line) refer to comparisons with the no-∆p control. In *panel d*, the square brackets refer to comparisons with the no-Na^+^ condition; ns indicates ‘not significant’.

NorM-VC and NorM-PS are homologous proteins that share 41% identical plus 18% similar residues and contain conserved catalytic carboxylates (Fig. 1c). To further investigate structural differences in the N- and C-lobes of NorM-VC and NorM-PS that might be responsible for the differences in ion coupling, chimaeric proteins were constructed. The coding sequence for the N-lobe of NorM-VC (residues 1–224) was fused to the coding sequence for the C-lobe of NorM-PS (residues 227–464) (Figs. 8a and 1c), yielding chimaeric *n*VC-*c*PS. The second chimaera (termed *n*PS-*c*VC) was constructed by fusing the coding sequence of the N-lobe of NorM-PS (residues 1–226) to that of the C-lobe of NorM-VC (residues 225–461) (Figs. 8a and 1c). The chimaeric proteins were expressed in the plasma membrane of *L. lactis* under identical experimental conditions and at expression levels that were in a comparable range as NorM-VC and NorM-PS (Fig. 8b and S3). The higher mobility of the monomer signal of NorM-PS compared to NorM-VC on SDS-PAGE is consistent with previous studies^20,22^. It is also observed for chimaeras containing the C-lobe of NorM-PS (*n*VC-*c*PS, and *n*VC-*c*PS-D36N) and is most likely due to increased SDS binding to the C-lobe of NorM-PS.

**Fig. 9:**
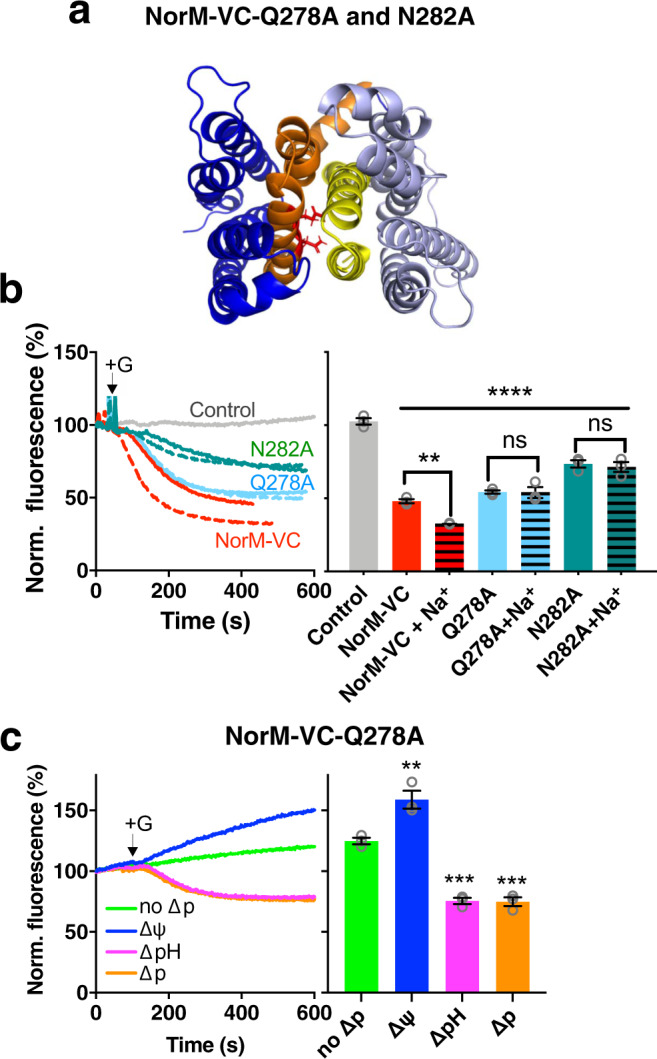
Na^+^ dependency of NorM-VC-mediated ethidium efflux requires a specific domain interface. **a** Mutations Q278A and N282A (TM8) (in red stick representation) are located at the interface between the N-lobe and C-lobe, formed by TM1-TM2 (yellow) and TM7-TM8 (orange), in inward-facing NorM-VC. **b** The mutations disable the stimulation by Na^+^ (dotted traces) of the ∆p-dependent ethidium efflux by wildtype NorM-VC in cells in buffer pH 7.0. The histograms show levels of ethidium fluorescence at 400 s in repeat experiments in the absence of Na^+^ (open bars) or presence of 1 mM Na^+^ (horizontally-striped bars). **c** Ethidium efflux in NorM-VC-Q278A-expressing cells in the absence of the ∆p (no ∆p, green trace) or the presence of ∆ψ (blue trace), ∆pH (pink trace), or ∆p (orange trace). Glucose (+G) and ionophores were added to the cells as described in Fig. 3c. These data demonstrate electroneutral ethidium^+^/H^+^ antiport for the Q278A mutant. Data represent observations in three experiments (*n* = 3) with independently prepared batches of cells. Values are expressed as mean ± s.e.m. (two-way analysis of variance; ****P < 0.0001, ***P < 0.001, **P < 0.01). Asterisks directly above bars or groups of bars (indicated by horizontal line) refer to comparisons with the non-expressing control (**b**) or no-∆p control (**c**). In **b**, the square brackets refer comparisons with the no-Na^+^ condition; ns indicates ‘not significant’.

The *n*VC-*c*PS and *n*PS-*c*VC transporters were both ethidium efflux-active. For each of the chimaeras, the efflux was strictly dependent on the presence of the ∆pH (interior alkaline), whereas no ethidium transport was seen in the presence of the ∆ψ only (Fig. 8c). Thus, both chimaeras mediate electroneutral ethidium^+^/H^+^ antiport analogous to NorM-VC-D36N (Fig. 5a) and wildtype NorM-PS (Fig. 6a). The inhibitory effect of D36N in the N-lobe of *n*VC-*c*PS suggests that D36 must remain dissociated for activity of this chimaera (Fig. 8c). On the other hand, the neutral response of D38N in the N-lobe of *n*PS-*c*VC (Fig. 8c) follows the observations for NorM-PS (Fig. 6b). Neither of the *n*VC-*c*PS and *n*PS-*c*VC transporters showed a response to 1 mM Na^+^ during ∆p-dependent ethidium efflux at pH 7.4, where Na^+^ has a 25,000-fold excess over H^+^ (Fig. 8d). This was corroborated in ethidium binding experiments with purified chimaeric proteins in which Na^+^ did not enhance the ethidium binding affinity (Fig. 4a). Clearly, Na^+^ coupling is lost when the N-lobe or C-lobe in NorM-VC is replaced by the corresponding lobe in NorM-PS, suggesting that the underlying mechanism employs specific communication between the native N- and C-lobes. To further test this notion, experiments focused on the amide residues Q278 and N282, which are located at the N-lobe: C-lobe interface in an inward-facing model of NorM-VC (Fig. 9a) and which are conserved in NorM-PS (Fig. 1c). The NorM-VC-Q278A and NorM-VC-N282A mutants were still ethidium transport-active, but the transport reaction was no longer stimulated by 1 mM Na^+^ (Fig. 9b). Further tests on the transport energetics of the Q278A mutant demonstrate electroneutral ethidium^+^/H^+^ antiport (Fig. 9c), and hence, the loss of a proton in the transport reaction compared to wildtype NorM-VC. These results indicate that electrogenic ethidium^+^/(H^+^, Na^+^) antiport and ethidium^+^/2H^+^ antiport in NorM-VC require a specific interface between the N-lobe and C-lobe.

## Discussion

**Fig. 10:**
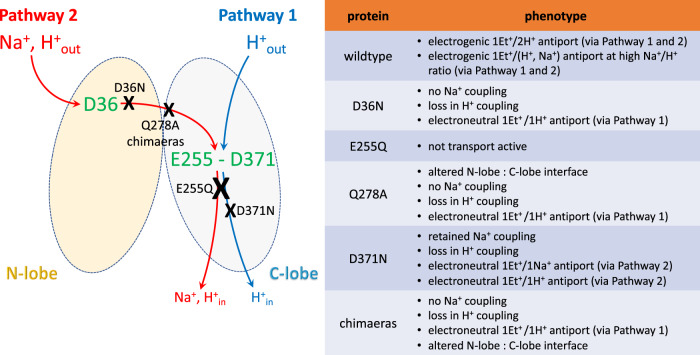
Organisation of the two ion translocation pathways in NorM-VC and the transport properties of mutants. H^+^ translocation in Pathway 1 involves E255 and D371 in the C-lobe. Promiscuous Na^+^ and H^+^ translocation in Pathway 2 requires D36 in the N-lobe and E255 in the C-lobe. Black crosses and text refer to inhibition (see ‘Discussion’).

In this work, we studied the mechanism of energy coupling in NorM-VC and obtained data that extend previous suggestions based on structural approaches. In particular, our results demonstrate the presence of two distinct ion-translocation pathways in NorM-VC. Pathway 1 is H^+^-selective, active in the C-lobe, and involves E255 and D371. Pathway 2 is promiscuous for Na^+^ and H^+^ as alternative coupling ions, starts at D36 in the N-lobe, and proceeds across the domain interface to E255 in the C-lobe. To facilitate a further discussion of the data, we refer to the schematic and summary of functional properties of mutants in Fig. 10.

The measurements of ethidium efflux as a function of the magnitude and composition of the ∆p indicate that wildtype NorM-VC mediates a ∆ψ-dependent (electrogenic) ethidium^+^/nH^+^ antiport reaction in which *n* ≥ 2 (Fig. 3c, d), whereas the D36N mutant exhibits ∆ψ-independent (electroneutral) ethidium^+^/H^+^ antiport (Fig. 5a). The simplest model that explains these observations is one where NorM-VC catalyses electrogenic ethidium^+^/2H^+^ antiport (*n* = 2). When the experiments were repeated in the presence of Na^+^, the Na^+^ dependency of energy coupling to the wildtype NorM-VC was found to increase with the alkalinity of the assay buffer. Furthermore, the D36N mutation was associated with a complete loss of the Na^+^ coupling, resulting in electroneutral ethidium^+^/H^+^ antiport (Fig. 5a). These results imply that the D36 pocket can use H^+^ or Na^+^ as alternative coupling ions depending on their availability, and that the Na^+^ coupling in NorM-VC causes a shift from electrogenic ethidium^+^/2H^+^ antiport to electrogenic ethidium^+^/(Na^+^, H^+^) antiport. In these experiments, Na^+^ could not be replaced by Rb^+^, Cs^+^ or Li^+^ (Fig. 4a). The Na^+^–H^+^ promiscuity of the D36 binding pocket is supported by our MD simulations of Na^+^ binding to outward-facing NorM-VC in a phospholipid bilayer (Fig. 7 and S1). These simulations show that the binding of Na^+^ over H^+^ at high Na^+^/H^+^ ratios originates from the inhibition of Na^+^ binding in the D36 pocket by protonation of D36. It should be noted that while the biochemical assays in this work measure the activity of populations of non-synchronised transporters over minutes, the molecular events underlying substrate and ion translocation in individual transporters occur in the nanosecond time scale. Therefore, the time scales used in our MD simulations are appropriate to model the transport reactions.

As a previous study on the NorM-VC homologue NorM-PS in *P. stutzeri* reported on H^+^-coupled drug transport^23^, we compared the energetics of both MATE transporters under identical experimental conditions. We observed that wildtype NorM-PS mediates electroneutral ethidium^+^/H^+^ antiport in a Na^+^-independent fashion in a buffer pH range of 6.0–8.0 (Fig. 6a) and exhibits ethidium binding in a Na^+^-independent fashion (Fig. 4a). Furthermore, our MD simulations show impaired Na^+^ binding in the D38 pocket of NorM-PS relative to the D36 pocket in NorM-VC (Fig. 7 and S1). Moreover, the introduction of the D38N mutation, which should disable H^+^ binding to and dissociation from D38, did not inhibit ethidium efflux by NorM-PS (Fig. 6b). Thus, the D38 pocket does not contribute to H^+^ or Na^+^ coupling in these experiments. A crystal structure for PfMATE (PDB-ID: 3VVP) shows deep binding of norfloxacin in the N-lobe, which causes conformational changes that impair the binding of coupling ions in the D41 pocket^11^. For NorM-VC and NorM-PS, it is interesting to note that the fluorescence anisotropy responses for ethidium binding in Fig. 4a fall into two groups. NorM proteins containing the N-lobe of NorM-VC (NorM-VC ± Na^+^, Rb^+^, Cs^+^ or Li^+^; NorM-VC mutants D36N, E255Q, and D371N ± Na^+^; and *n*VC-*c*PS chimaera ± Na^+^) have a high mean fluorescence anisotropy value of 0.139 ± 0.004 at 1 µM protein, whereas the NorM proteins containing the N-lobe of NorM-PS (NorM-PS ± Na^+^ and the *n*PS-*c*VC ± Na^+^) have a significantly lower mean value of 0.096 ± 0.006 (*P* < 0.0001 in unpaired two-tailed student *t*-tests). These differences indicate that the interaction of NorM proteins with ethidium is, at least in part, dictated by the N-lobe. If so, ethidium binding to NorM-PS might impair the interactions of coupling ions in the D38 pocket similarly as reported for D41 pocket in PfMATE. As D36 in NorM-VC is functional in electrogenic ethidium^+^/(H^+^, Na^+^) antiport (Fig. 5a), the molecular details of ethidium binding in the N-lobes of NorM-VC and NorM-PS must be different. A previous study on 4,6-diamidino-2-phenylindole (DAPI) transport by NorM-PS reported electroneutral DAPI^2+^/2H^+^ antiport with a role of D38 in proton coupling^23^. The energetics of drug transport by NorM-PS is, therefore, drug dependent suggesting that the contributions of catalytic carboxylates to drug/ion antiport in MATE transporters can be affected by different binding mechanisms of drugs.

Given the differences in the energetics of ethidium transport by NorM-PS and NorM-VC, a set of chimaeric proteins was generated in which the N-lobe of NorM-PS was fused with the C-lobe of NorM-VC, and vice versa. The active ethidium efflux by the chimaeras with wildtype domains (*n*PS-*c*VC and *n*VC-*c*PS) is based on Na^+^-independent, electroneutral H^+^/ethidium^+^ antiport (Fig. 8c, d). This activity is similar to that of NorM-PS and NorM-VC-D36N, suggesting that these chimaeras lack ion-coupling via the N-lobe and must have retained H^+^ translocation in the C-lobe. This pathway (Pathway 1 in Fig. 10) contains two acidic side chains E255 and D371 in NorM-VC. Proteins structures of NorM-VC and homologues (PDB-ID: 3MKT, 3MKU and 4HUL) suggest that the two carboxyl residues form a dyad of ionisable side chains that mediate ion binding^26–28^.

In the measurements of ethidium binding to NorM proteins by fluorescence anisotropy, high-affinity binding in wildtype NorM-VC is Na^+^ dependent (Fig. 4a). The Na^+^ stimulation of ethidium binding was lost for E255Q in the C-lobe and D36N in the N-lobe (Fig. 4a), suggesting Na^+^ binding near these locations in the wildtype protein. Given the essential role of D36 at the external side of NorM-VC in Na^+^-coupled transport, both pockets must be part of a single Na^+^ conduction pathway, designated Pathway 2 in Fig. 10, in which the Na^+^ ions first enter the pathway via D36 and then move across the N-lobe: C-lobe interface to E255 in the C-lobe. The existence of Pathway 2 is supported by our MD simulations. Whereas the MD simulations with the outward-facing conformation of NorM-VC indicate that Na^+^ binds in the D36 pocket, the simulations with the inward-facing conformation point to the existence of a lateral Na^+^ permeation pathway from D36 to E255 across the interface between the N-lobe and C-lobe via (i) D36, Y133 and T200, (ii) A55, and (iii) T30 (Fig. S4). As the D371N mutant retains electroneutral Na^+^-coupled ethidium transport^20^, Na^+^ binding in the E255–D371N region is supported by both a carboxyl side chain and amide side chain at position 371, although the latter does inhibit ethidium binding (Fig. 4a), potentially due to loss in Na^+^ binding affinity. Changes in the interface between the N-lobe and C-lobe in NorM-VC, through the introduction of a Q278A or N282A mutation, result in the loss of Na^+^-coupled transport (Fig. 9b). Due to this loss, electrogenic ethidium/(H^+^, Na^+^) antiport for wildtype NorM-VC shifts to electroneutral ethidium/H^+^ antiport for the Q278A mutant (Fig. 9c) via Pathway 1. The N-lobe: C-lobe interface is also altered in the chimaeric proteins, which have phenotypes similar to the Q278A and N282A mutants with a functional Pathway 1 only (Fig. 8).

Several conclusions can be drawn from these data (Fig. 10). First, Pathway 2 can translocate H^+^, or Na^+^ at high Na^+^/H^+^ ratio (Fig. 3). Together with H^+^ translocation through Pathway 1, this leads to electrogenic ethidium^+^/(1H^+^,1H^+^) antiport and electrogenic ethidium^+^/(1H^+^,1Na^+^) antiport, respectively. Second, electroneutral ethidium^+^/H^+^ antiport in NorM-VC-D36N (Fig. 5a) is based on proton translocation via Pathway 1. Third, at a high Na^+^/H^+^ ratio, NorM-VC-D371N mediates electroneutral ethidium^+^/Na^+^ antiport without detectable input from H^+^ coupling^20^. The mutant also mediates residual ethidium^+^/H^+^ antiport in the absence of Na^+^ (Fig. 5b). Hence, Na^+^ and H^+^ translocation in the D371N mutant proceed via Pathway 2 in which H^+^ and Na^+^ compete. Pathway 1 might not be functional in this mutant. Fourth, as E255 is essential in Pathway 1 and 2, the E255Q mutant cannot mediate active ethidium efflux^20^ due to the simultaneous inhibition of both pathways. Therefore, the relative contributions of E255 and D371 to ion translocation are unequal. Finally, Na^+^ binding to NorM-VC stimulates ethidium binding (Fig. 4a), whereas ethidium binding is promoted at alkaline pH (Fig. 4b) and associated with proton release^20^. Therefore, Pathway 1 and 2 might facilitate different steps in the transport cycle. In agreement with this notion, the MD simulations demonstrate that protonation of D371 prevents reversed flow of Na^+^ back into the Na^+^ translocation pathway following the release of the sodium ion in the cytosol (Fig. S4).

In summary, using engineered chimaeric and mutant MATE multidrug transporters, this work demonstrates in well-established functional assays that NorM-VC contains two distinct ion translocation pathways. These pathways differ in their ion selectivity and organisation in the N- and C-lobe. Our results extend previous proposals, based on structural analyses of NorM-VC and NorM-NG, of Na^+^ coupling in the C-lobe only. With contributions of both lobes to ion coupling, NorM-VC is more versatile than members of the DinF and eMATE subfamilies that rely on H^+^ coupling in the N-lobe or C-lobe, respectively. This conclusion is also supported by the distribution of the catalytic carboxylates, which are present in both the N- and C-lobe in NorM-VC but are restricted to single lobes in other MATE subfamilies. The Na^+^/H^+^ promiscuity of NorM-VC provides flexibility in energy coupling with fluctuations in salinity in the natural habitats of *V. cholerae*. This is relevant in transitions of the bacterium between fresh water and body fluids, and in locations where reservoirs of fresh and brackish water mix at estuaries between river environments and maritime environments^29,30^.

## Methods

### Bacterial strains

Drug-hypersensitive *Lactococcus lactis* NZ9000 Δ*lmrA* Δ*lmrCD*^31^, harbouring pNZ8048-derived plasmids with an inducible *nisA* promoter^32^, was grown at 30 °C in M17 medium (Oxoid and Formedium) supplemented with 25 mM glucose and 5 µg/mL chloramphenicol. For cloning in pET19b-derived plasmids, *E. coli* DH5*α* was grown in Luria-Bertani Broth medium supplemented with 100 µg/ml carbenicillin. *L. lactis* strains expressing NH_2_-terminal His_10_-tagged wildtype NorM-VC or the D36N, E255Q and D371N mutants were described in an earlier publication^20^.

### Site-directed mutagenesis and DNA cloning

For the generation of mutants, the QuickChange method was applied as per the manufacturer’s instructions (Agilent Technologies) with primers listed in Table S1. Alternatively, the ‘Round-the-Horn’ method was used with two non-overlapping primers (Table S1), one of which contains the desired mutation at 5’ end. The primers were phosphorylated using T4 polynucleotide kinase (Thermo Fisher Scientific) as per product manual and directly used in the mutagenic PCR reactions. A typical 50 µl PCR reaction consisted of 1× buffer provided with the Phusion DNA polymerase kit (Thermo Fisher Scientific), 0.2 mM dNTPs, 10 µM forward and reverse primer, 10 ng plasmid DNA template and 1 unit of Phusion DNA polymerase. The PCR cycling parameters were 98 °C for 1 min, (98 °C for 15 s, 55 °C for 30 s, 72 °C for 30 s/kb) × 20–25 cycles, 72 °C for 5 min. The amplified product was detected using agarose gel electrophoresis. After PCR amplification, parental DNA was digested using FastDigest DpnI (Thermo Fisher Scientific). Undigested DNA was gel purified and ligated (optional in case of QuickChange). The DNA was finally used to transform chemically (CaCl_2_) competent *E. coli* DH5α cells.

For cloning of *norM-PS*, the coding sequence (GenBank ID EHY79494.1) was optimised for expression in *L. lactis* using the online codon optimisation tool from Integrated DNA Technology (IDT). A NdeI restriction site and coding region for an NH_2_-terminal His_10_-tag were added to the 5’-terminus, and an XhoI restriction site was added to the 3′ terminus. The synthesised gene was digested using NdeI and XhoI restriction enzymes. Using the same restriction sites in pET19b harbouring the *norM-VC* gene^7^, the original insert was replaced by the *norM-PS* gene. Prior to subcloning into the lactococcal pNZ8048, the insert DNA was sequenced at Eurofins Genomics. The *norM-PS* gene was excised from pET19b-NorM-PS using NcoI and BamHI restriction sites and was ligated into digested pNZ8048 vector (derived from pNZ-NorM-VC^20^), after which the DNA was used to transform electrocompetent *L. lactis* cells. The subcloned gene was again sequenced to confirm the original DNA sequence.

The NorM-VC and NorM-PS chimaeras were generated using the FastCloning method^33^ and the primers listed in Table S1. Chimaera *n*VC-*c*PS refers to the protein consisting of the N-lobe of NorM-VC (amino acid residues 1–224) and the C-lobe of NorM-PS (amino acids residues 227–464), whereas chimaera *n*PS-*c*VC refers to the N-lobe of NorM-PS (amino acids 1–226) and the C-lobe of NorM-VC (amino acid residues 225–461). For the generation of chimaera *n*VC-*c*PS, the primer pair NorM-VC N-lobe (fw and rev) was used to amplify the N-lobe of NorM-VC alongside the rest of the *E. coli* pET19b-NorM-VC plasmid in one PCR reaction. The primer pair NorM-PS C-lobe (fw and rev) was used to amplify the C-lobe of NorM-PS in a separate PCR reaction. Each 50 µl PCR reaction mixture consisted of 1× HF buffer (provided with DNA Phusion polymerase kit), 0.2 mM dNTPs, 5 picomoles of each primer, 10 ng plasmid DNA template (pET19b-NorM-VC and pET19b-NorM-PS) and 1 unit of Phusion DNA polymerase. The PCR cycling parameters were 98 °C for 1 min, (98 °C for 15 s, 55 °C for 30 s, 72 °C for 30 s/kb) × 18 cycles, 72 °C for 5 min. The product was confirmed using agarose gel electrophoresis. After PCR amplification, 1 µl of DpnI was added to the PCR products and the two reaction mixtures were pooled together in the ratios 1:1, 1:2, 1:4 and incubated at 37 °C for 1 h to digest the template DNA. 2 µl of the digested product was used to transform competent *E. coli* DH5α cells. Cloned DNA was sequenced to confirm the fusion was obtained as intended. The chimaera *n*PS-*c*VC was generated in the same manner as described for *n*VC-*c*PS using the primer pair NorM-PS N-lobe and NorM-VC C-lobe (Table S1).

### NorM protein expression in cells

M17 growth medium containing 25 mM glucose and 5 µg/ml chloramphenicol was inoculated with 2.5% overnight culture and grown to an OD_660 nm_ of 0.5–0.6 at 30 °C. Protein expression was induced by addition of 0.1% (v/v) of the culture supernatant of the nisin A-producing strain *L. lactis* NZ9700^32^, which results in a final concentration of ~10 pg nisinA/ml. Further increase in the amount of nisin does not lead to enhanced NorM protein expression. Cells were further grown for 1 h and were collected by centrifugation at 12,300 × *g* for 12 min at 4 °C (Sorvall Evolution RC, SLC-6000 rotor). The pellet was resuspended in ice-cold 100 mM potassium phosphate (KP_i_) buffer (pH 7.0) and centrifuged at 4200 × *g* for 30 min at 4 °C. The presence of NorM proteins in membrane vesicles was assessed on western blot probed with mouse anti-polyhistidine tag antibody (Sigma-Aldrich, cat. no.: H1029) and goat antimouse antibody (Sigma-Aldrich, cat. no.: A4416).

### Bioenergetic considerations

The choice of lactococcal cells in these drug transport studies is useful for two reasons. First, *L. lactis* is a simple fermentative organism that generates ATP by substrate-level phosphorylation during (i) glucose uptake and phosphorylation by the phosphoenolpyruvate: carbohydrate phosphotransferase system and (ii) subsequent metabolism of the glucose-6P in the glycolytic pathway^34^. The bacterium uses this ATP pool for a variety of processes that include proton extrusion across the plasma membrane by the F_1_F_0_ H^+^-ATPase. The outwardly-directed translocation of protons and their positive charge results in the generation of two components: a chemical proton gradient (ΔpH, inside alkaline) and a membrane potential (Δψ, interior negative). These components impose an inwardly-directed force on the protons referred to as the proton motive force (Δp), which is expressed in the equation Δp = Δψ − ZΔpH (mV). In this equation, Z = 2.3RT/F (where R is the gas constant, T the absolute temperature and F the Faraday constant) and equals 59.1 mV at 25 °C^35–37^. By catalysing proton/drug antiport, MATE transporters mediate proton uptake, which consumes the Δp. Proton uptake by secondary-active membrane transporters and proton extrusion by the primary-active F_1_F_0_ H^+^-ATPase occur simultaneously and contribute to a chemiosmotic proton circuit^36^ that sustains proton circulation in the cell^37^. Although proton circulation plays a central role in energy transduction in *L. lactis*, sodium circulation also contributes and is supported by the presence of Na^+^ in the M17 growth medium. The sodium motive force (Δp_Na_, interior negative and low) is defined in an analogous way (Δp_Na_ = Δψ − ZΔpNa), and is generated from the Δp by electrogenic H^+^/Na^+^ antiport activity across the lactococcal plasma membrane. In transport assays with washed cells in KPi buffer, sodium circulation in cells is dependent on the addition of Na^+^ to the buffer.

Second, *L. lactis* makes high demands on the nutritional composition of its growth medium and does not generate typical bacterial intracellular energy reserves such as polyhydroxybutyrate or polyglucan. A short incubation of the cells with a protonophore that dissipates the Δp, therefore, causes rapid depletion of the intracellular ATP pool due to compensatory H^+^ efflux by the F_0_F_1_ H^+^-ATPase^38^. In our previous work, we established that NorM-VC mediates ethidium uptake (via ethidium/ion antiport) in de-energised cells upon imposition of an inwardly directed chemical ethidium gradient across the plasma membrane (exterior high). We also observed drug uptake by multidrug transporters in the ABC and MFS families under these conditions^38–42^. We frequently use this method to preload cells with ethidium and establish an intracellular ethidium pool, prior to initiating ethidium efflux by glucose addition. The metabolism of glucose allows the cells to again generate ATP and Δp, as well as a substantial Δp_Na_ when Na^+^ is present in the external buffer at a sufficient concentration^38^.

### Ethidium transport measurements

Ethidium is a lipophilic monovalent cation that, in contrast to many antibiotics, lacks moieties that can accept or donate protons in the physiological pH range^43^. Therefore, ethidium is ideal for studying energy coupling in secondary-active multidrug transporters that contain separate translocation pathways for H^+^ and substrates. To prepare cells for ethidium transport measurements, the cells were harvested by centrifugation and washed in ice-cold wash buffer (50 mM KPi, 5 mM MgSO_4_, pH 7.0) by centrifugation at 3000 × *g* for 10 min. The cell pellet was resuspended in wash buffer supplemented with 0.5 mM of the uncoupler 2,4-dinitrophenol^20^ and incubated at 30 °C for 40 min. To remove the uncoupler, the de-energised cells were washed thrice with wash buffer by centrifugation at 3000 × *g* for 10 min, and resuspended in this buffer to OD_660_ of 5.0. In the ethidium transport assay, the cells were diluted 10 times in 2 ml of wash buffer (unless stated otherwise) in a 3 ml glass cuvette at 30 °C. After 30 s, 2 µM ethidium bromide was added, and fluorescence was recorded. After reaching saturation in fluorescence, 12.5 mM glucose was added to energise cells and to initiate ethidium efflux. Where indicated, valinomycin and/or nigericin were added 3 min before the glucose at a concentration of 0.1 μM and 0.5 µM, respectively. For ethidium transport assays in K^+^-free Tris buffer, the wash buffer for DNP-treated cells was 20 mM Tris-Cl (pH 7.0) containing 5 mM MgSO_4_. Fluorescence measurements were performed in a Perkin Elmer LS-55B Luminescence spectrometer at λ_ex_ of 500 nm and λ_em_ of 580 nm with slit widths of 5 and 10 nm, respectively. Figures contain representative traces obtained in 3 or more independent experiments with separate batches of cells. Histograms show mean levels of ethidium fluorescence in the repeat experiments.

### Membrane potential measurements

Cells were prepared as described under ‘Ethidium transport measurements’ and were diluted 10-fold in 50 mM KPi supplemented with 5 mM MgSO_4_ at pH 6.0, 7.0, and 8.0 to attain an OD_660_ = 0.5 in a glass cuvette. The generation of metabolic energy in the cells was initiated by the addition of 12.5 mM glucose and incubation for 3 min. DiOC_2_(3) probe (Thermo Fisher Scientific) was added to a concentration of 10 µM after which fluorescence was monitored at λ_ex_ = 488 nm and λ_em_ = 620 nm with slit widths of 5 nm and 10 nm, respectively. When steady-state fluorescence was achieved, 0.5 µM nigericin was added to allow conversion of the ∆pH into a ∆ψ. After the establishment of a new steady-state, 0.5 µM valinomycin was added to dissipate the proton-motive force completely. As NorM-VC activity might affect DiOC_2_(3) partitioning across the membrane, the fluorescence measurements were performed in non-expressing control cells.

### Preparation of inside-out vesicles and protein purification

Inside-out oriented plasma membrane vesicles were prepared from lactococcal cells as described^20^ with modifications. Cell pellets collected from 1 L culture were resuspended in 20 ml KPi (pH 7.0) containing 5 mg/mL lysozyme (from chicken egg white) and a tablet of Complete-Protease inhibitor cocktail (Sigma-Aldrich) and incubated for 30 min at 30 °C. Cell lysis was performed by passing the cell suspension 3 times through a Basic Z 0.75 kW Benchtop Cell Disruptor (Constant Systems, Northlands, UK) at 20,000 psi. The suspension containing the lysed cells was supplemented with 10 µg/mL DNase (Sigma-Aldrich) and 10 mM MgSO_4_ and incubated for 30 min at 30 °C. Following the addition of 15 mM K-EDTA (pH 7.0), the suspension was centrifuged at 4 °C for 40 min at 14,000 × g (Sorvall Evolution RC-6 PLUS, F21S rotor). The supernatant containing the membrane vesicles was retained and ultra-centrifuged at 125,000 × g for 50 min at 4 °C (Beckman Type-50.2-Ti rotor). Cell pellets were resuspended in 50 mM KPi (pH 7.0) containing 10% glycerol, and stored in liquid nitrogen. Protein concentration was determined by Bio-Rad DC protein assay kit using bovine serum albumin as the standard.

NorM proteins were purified from inside-out membrane vesicles using metal ion-affinity chromatography. Membrane vesicles (5 mg membrane protein/ml) were solubilised for 4 h with mild shaking at 4 °C in 50 mM KPi (pH 8.0), 10% (v/v) glycerol, 0.1 M KCl and 1% (w/v) n-dodecyl-β -D-maltopyranoside (DDM). Non-solubilised membrane vesicles and cell debris were removed by centrifuging the suspension at 208,000 × *g* (Beckman Type-70.1-Ti rotor) at 4 °C for 30 min. Nickel-nitrilotriacetic acid resin (Ni-NTA) was equilibrated by washing thrice with 5 resin volumes of ultrapure water, and twice with 5 resin volumes of wash buffer A (50 mM KPi (pH 8.0), 0.1 M KCl, 10% (v/v) glycerol, 0.05% (w/v) DDM and 20 mM imidazole) at a ratio of 10 mg His-tagged protein/mL of resin. The suspension was left on a rotating wheel for overnight binding at 4 °C. The resin was transferred to a 2 mL volume Biospin disposable chromatography column (Bio-Rad). After subsequent washing with 20 volumes of wash buffer A and 30 volumes of wash buffer B (50 mM KPi (pH 7.0), 0.1 M KCl, 10% (v/v) glycerol, 0.05% (w/v) DDM and 20 mM imidazole), the His-tagged protein was eluted with 3–4 volumes of elution buffer (50 mM KPi (pH 7.0), 0.1 M KCl, 5% (v/v) glycerol, 0.05% (w/v) DDM and 150 mM imidazole). The eluted purified protein was kept on ice and immediately used in experiments. Purified protein was quantified in the Micro BCA protein assay (Thermo Fisher Scientific) using bovine serum albumin as the standard. The purity of the protein was checked on 10% SDS-PAGE stained with Coomassie Brilliant Blue.

### Measurements of ethidium binding

To measure ligand binding by fluorescence anisotropy^44^, increasing amounts of purified protein were added from a 0.4 mg/ml stock solution in 2 ml of 50 mM KPi buffer (pH 7.0) containing 0.2 mM KCl, 0.05% DDM and 2 µM ethidium bromide, to final protein concentrations ranging from 0.036 to 1 µM. To test the effect of Na^+^ on ethidium binding, 0.5 mM Na_2_SO_4_ was included in the buffer. The effect of the proton concentration on ethidium binding was tested by varying the pH of the buffer from 6.0 to 9.0 in steps of 0.2 through the addition of concentrated KOH. The protein elution buffer (see above) was used as a negative control to generate the baseline. The fluorescence anisotropy was measured in a Perkin Elmer LS-55B Luminescence spectrometer at λ_ex_ of 500 nm and λ_em_ of 580 nm with slit widths of 10 nm each for samples equilibrated for about 2 min. The data in Fig. 4a, b were fitted to a two-sites specific-binding model and biphasic dose-response model, respectively, in GraphPad Prism v8.4.3.

### MD simulations

The MD simulations were performed using GROMACS v2018.4^45^. A summarising schematic of all MD simulations is presented in Fig. S5. The all-atom Fuji force field^46,47^ was used for protein, lipids and ions, with TIP3P water^48^. The constant temperature was set at 303 K using a Nosé–Hoover thermostat^49,50^. Constant pressure was set at 1 bar using the semi-isotropic Berendsen barostat^51^ or Parrinello–Rahman barostat^52^ with a coupling constant of 2.0 ps for both barostats. Detailed simulation conditions and methods were as described in an earlier publication^53^ in which electrostatic interactions were calculated using particle mesh Ewald (PME) method^54^ with a real-space cut-off of 1.0 nm. Lennard-Jones interactions were calculated using Lennard-Jones particle mesh Ewald (LJ-PME) method^55^ with geometric approximations of the combination rules in reciprocal space. The Verlet cut-off scheme was used for the neighbour list. The linear constraint solver (LINCS) algorithm with lincs-order of 6 was used to constrain all bonds.

As initial structures for MD simulations, we used the crystal structure of the outward-facing conformation of NorM-VC (PDB-ID: 3MKT) and a homology model of NorM-PS based on this structure^22^. Furthermore, the initial structures of inward-facing NorM-VC and NorM-PS were based on homology models built using the inward-facing structure of PfMATE (PBD-ID: 6FHZ). All homology models were calculated by the SWISS-MODEL homology server (https://swissmodel.expasy.org/) and were validated with MolProbity v4.4 (http://molprobity.biochem.duke.edu/) following optimised hydrogen placement and all-atom contact analysis complemented by updated versions of covalent-geometry and torsion-angle criteria.

The topology files of proteins and lipids were generated by the GROMACS pdb2gmx subprogram with the hydrogen virtual site option. Three protonation states of D36, E255 and D371 in NorM-VC, all unprotonated (designated ‘n’), D371 protonated (designated ‘d’), and E255 protonated (designated ‘e’) were tested for inward-facing NorM-VC (*vin*, *vid* and *vie*) and outward-facing NorM-VC (*von*, *vod* and *voe*). Equivalent states were studied for inward-facing NorM-PS (*pin*, *pid* and *pie*) and outward-facing NorM-PS (*pon*, *pod* and *poe*), giving a total of 12 systems. A lipid bilayer modelled for *E. coli* and consisting of 8 types of phosphatidylethanolamine and phosphatidylglycerol (173 molecules of PMPE [1-palmitoyl-2-cis-9,10-methylene-hexadecanoic-acid-sn-glycero-3-phosphoethanolamine]; 50 molecules of PMPG [1-palmitoyl-2-cis-9,10-methylene-hexadecanoic-acid-sn-glycero-3-phosphoglycerol]; 70 molecules of PVPE [1-palmitoyl-2-vacenoyl-sn-glycero-3-phosphatidylethanolamine]; 16 molecules of PVPG [1-palmitoyl-2-vacenoyl-sn-glycero-3-phosphatidylglycerol]; 38 molecules of QMPE [1-pentadecanoyl-2-cis-9,10- methylene-hexadecanoic-acid-sn-glycero-3-phosphoethanolamine]; 35 molecules of PYPE [1-palmitoyl-2-palmitoleoyl-sn-glycero-3-phosphatidylethanolamine]; 21 molecules of PYPG [1-palmitoyl-2-palmitoleoyl-sn-glycero-3-phosphatidylglycerol]; 33 molecules of VYPE [1-vacenoyl-2-palmitoleoyl-sn-glycero-3-phosphatidylethanolamine]) was generated using the CHARMM-GUI^56^. This lipid composition was based on published LC/ESI-MS/MS analyses of the plasma membrane of *E. coli*^57^. The lipid bilayer was hydrated with 0.15 M NaCl electrolyte. With the PME and LJ-PME methods, the energy of the bilayer system was minimised using alternating steepest-descent and conjugate gradient methods. After minimisation, the system was equilibrated initially for 1 ns using semi-isotropic Berendsen barostat and later for 1000 ns using semi-isotropic Parrinello–Rahman barostat with a time step of 5 fs (Fig. S5a).

The initial protein structures were embedded in the equilibrated lipid bilayers at 1000 ns using LAMBADA^58^ and overlapping lipids whose heavy atoms were within 0.12 nm of the heavy atoms of the protein were removed (Fig. S5a). The energy of the system was minimised through the same procedure as the bilayer, followed by isothermal-isobaric ensemble equilibration using semi-isotropic Parrinello–Rahman barostat with position restraints only on the heavy atoms of the protein and not on the heavy atoms of lipids, initially for 1 ns with a time step of 2 fs, later for 200 ns with a time step of 5 fs. Using the final structures at 200 ns as initial structure, four unconstrained simulations were carried out initially with a time step of 2 fs for 50 ns with a different initial velocity, that satisfy a Maxwell-Boltzmann distribution at 303 K. Then the production runs were carried out for 500 ns with a time step of 5 fs, and used for analysis. The calculated RMSD value during production runs of the protein models in 5 independent simulations quickly moved towards the same plateau of approx. 5 Å for outward-facing NorM-PS. A similar RMSD profile, with a stable plateau value of close to 2.5 Å, is obtained for the second homologue model in our study, of inward-facing NorM-VC. For the crystal structure of outward-facing NorM-VC, an RMSD plateau value of 4 Å was reached. The RMSD values indicate that the protein conformations are all stable in silico as would be observed for a real membrane protein structure.

The following MD simulations were performed to study the interaction of Na^+^ in the D36-D38 pockets in outward-facing NorM proteins (Fig. S5b): *(simulation i)* The final structures at 500 ns of equilibrated NorM-VC (*von*) and NorM-PS (*pon*) were modified so that the water molecule closest to D36 and D38 was replaced with a Na^+^ ion. To test whether the Na^+^ was retained in the pocket over time, four 100 ns equilibrium simulations were carried out with a time step of 2 fs with different initial velocities for the NorM-VC and NorM-PS systems. Using GROMACS tool gmx energy, interaction energies between the Na^+^ and surrounding residues within 0.8 nm distance were calculated. The interaction energies were averaged over four MD simulations from 90 to 100 ns. *(simulation ii)* The simulations on Na^+^ retainment were performed from 0 to 100 ns for outward-facing NorM-VC (*von*) containing protonated D36. (*simulation iii*) For MD simulations of direct binding of Na^+^ in the D36-D38 pockets, four 500 ns trajectories were determined for NorM-VC (*vod*), in which Na^+^ binding was observed, and for the equivalent NorM-PS (*pod*). The minimum distances between Na^+^ in the external environment and D36-D38 were calculated using the GROMACS tool gmx mindist. MD simulations of direct binding of Na^+^ in the D36-D38 pockets were also performed for all 12 systems of inward-facing and outward-facing NorM-VC and NorM-PS using protocols analogous to simulation iii above.

To study the Na^+^ permeation pathway in inward-facing NorM-VC, the Na^+^- bound structure of one of four simulations of NorM-VC (*vin*) at 500 ns served as the initial structure for four 100 ns-simulations with a time step of 2 fs and with a different initial velocity (Fig. S5c). Subsequently, the production runs were carried out for 100 ns with a time step of 4 fs. For one of four obtained trajectories, the bound Na^+^ to D36 was specified at 0 ns, then the minimum distance between the Na^+^ and D36 was calculated using the GROMACS tool gmx mindist. The minimum distance between Na^+^ and E255 was calculated for the trajectory in inward-facing NorM-VC (*vid*) over 1 µs, during which trapping of Na^+^ near E255 was observed.

### Statistics and reproducibility

The data points in whole cells studies and fluorescence anisotropy measurements were obtained in three or more experiments using independent batches of cells and purified proteins. The significance of data was tested by ANOVA. Asterisks directly above bars or groups of bars (indicated by horizontal line) in the histograms refer to comparisons with the control (e.g. no-∆p condition or non-expressing cells); asterisks above square brackets refer to specific comparisons; *P < 0.05; **P < 0.01; ***P < 0.001; ****P < 0.0001; ns, not significant. Specific details are included in the figure legends.

### Reporting summary

Further information on research design is available in the Nature Research Reporting Summary linked to this article.

## Supplementary information

Supplementary Information

Reporting Summary

## Data Availability

Data that support the findings of this study have been deposited in the University of Cambridge research repository Apollo with DOI link 10.17863/CAM.64600 (ref. ^59^) or are available from the corresponding author upon reasonable request.
