# Supplementary material for: Engineered MATE multidrug transporters reveal two functionally distinct ion-coupling pathways in NorM from Vibrio cholerae
Source: Commun Biol. 2021 May 11;4:558. doi: 10.1038/s42003-021-02081-6 (PMC8113278; doi:10.1038/s42003-021-02081-6)
Supplement: Supplementary file 1 — Supplementary Information [file 42003_2021_2081_MOESM1_ESM.pdf]

SUPPLEMENTARY INFORMATION

Engineered MATE multidrug transporters reveal two functionally distinct ion-coupling pathways in NorM from *Vibrio cholerae*

Sagar Raturi,[1]†‡ Asha V. Nair,[1]† Keiko Shinoda,[2] Himansha Singh,[1] Boyan Bai,[1] Satoshi Murakami,[3] Hideaki Fujitani,[4]§ Hendrik W. van Veen[1]*

[1]Department of Pharmacology, University of Cambridge, Tennis Court Road, Cambridge CB2 1PD, UK

[2]Microbial Membrane Transport Engineering, Biotechnology Research Center, The University of Tokyo, 1-1-1 Yayoi, Bunkyo-ku, Tokyo 113-8657, Japan

[3]Department of Life Science, Tokyo Institute of Technology, Nagatsuta, Midori-ku, Yokohama 226-8501, Japan

[4]Laboratories for Systems Biology and Medicine, Research Center for Advanced Science and Technology, The University of Tokyo, 4-6-1 Komaba, Meguro-ku, Tokyo 153-8904, Japan

† these authors contributed equally to this work

‡ Present address: University College Dublin Clinical Research Centre, St. Vincent's University Hospital, Elm Park, Dublin 4, Dublin, Ireland D04 T6F4

§This paper is dedicated to the memory of our colleague Professor Hideaki Fujitani, who recently passed away.

*Corresponding author. Email: hwv20@cam.ac.uk (H.W.v.V.)

Table S1. Primers used in site-directed mutagenesis and the construction of *n*VC-*c*PS and *n*PS-*c*VC chimaeras.

**Site-directed mutagenesis**		
**Mutation**	**Method**	**Primer sequence**
NorM-VC N282A	QuickChange	Fw: aaacaccaaagaagagaaagcgagcgccacttggtgtgcc
		Rev: ggcacaccaagtggcgctcgctttctcttctttggtgttt
NorM-VC Q278A	QuickChange	Fw: aaattgagcgccactgcgtgtgccgccacgac
		Rev: gtcgtggcggcacacgcagtggcgctcaattt
NorM-PS D38N	Round-the-horn	Fw: aatacagtaatggccggtcgtg
		Rev: gacaaatcccatagcggtactggc
NorM-PS E257Q	Round-the-horn	Fw: caggcatctatctttagcgttatagcac
		Rev: tgcgaagacagcaactccaatcg
NorM-PS D373N	Round-the-horn	Fw: aatgtagtacaggtgacggctg
		Rev: gctgaactggaaaagagcggc

**Construction of *n*VC-*c*PS and *n*PS-*c*VC chimaeras**	
NorM-VC N-lobe	Fw: tacatgtgccagacgcttagagg
	Rev: ctcgaggatccggctgc
NorM-VC C-lobe	Fw: aaagtgtttgagactttccataaaccac
	Rev: atgcttgtagtggtcgtcgatatg
NorM-PS N-lobe	Fw: cgaccactacaagcatatgcctacagagttgcgtttacg
	Rev: aaagtctcaaacactttgctggcttggtacgctgg
NorM-PS C-lobe	Fw: cctctaagcgtctggcacatgtacaattattttgccaatttgaatggc
	Rev: gcagccggatcctcgagttaaacgatagctcgtctaagaacag

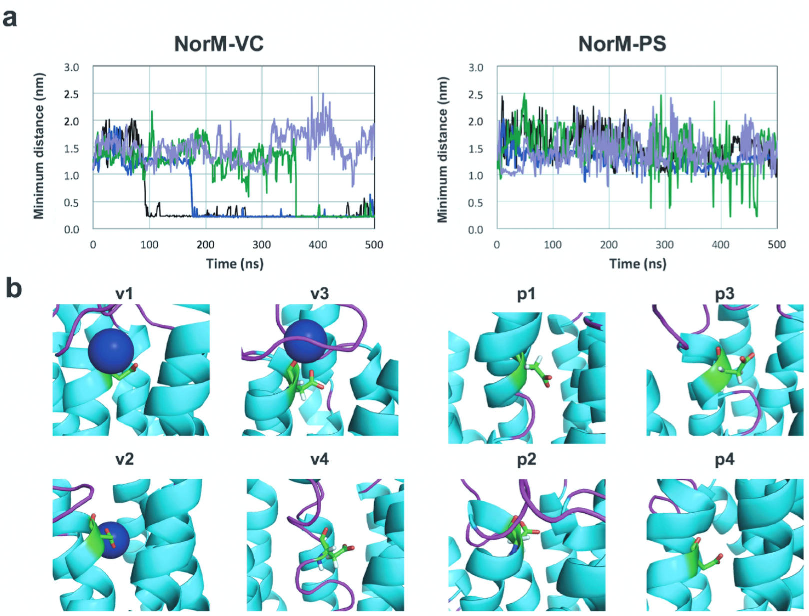

Figure S1. **Na^+ binding to the D36/D38 pockets in outward-facing NorM-VC and NorM-PS.** The interaction of Na^+ with the D36 and D38 pockets was compared by simulating the movement of the free ion into the empty pockets over a time span of 500 ns. (**a**) Minimum distance between Na^+ and D36/D38 is plotted against the simulation time. The minimum distance is defined as the distance between D36 or D38 and the nearest Na^+ among all Na^+ ions in the external environment. Four MD runs (v1 to v4 for NorM-VC and p1 to p4 for NorM-PS) are represented in different colours. The distances in three out of four runs for NorM-VC reached close to 0.25 nm and were maintained over time, indicating that Na^+ was bound close to D36. However, no binding of Na^+ in the D38 pocket was observed in the four simulations with NorM-PS. (**b**) Close-up views of the region close to D36 in NorM-VC or D38 in NorM-PS at 500 ns are presented for each of the four runs. The D36/D38 residues are indicated in a stick representation.

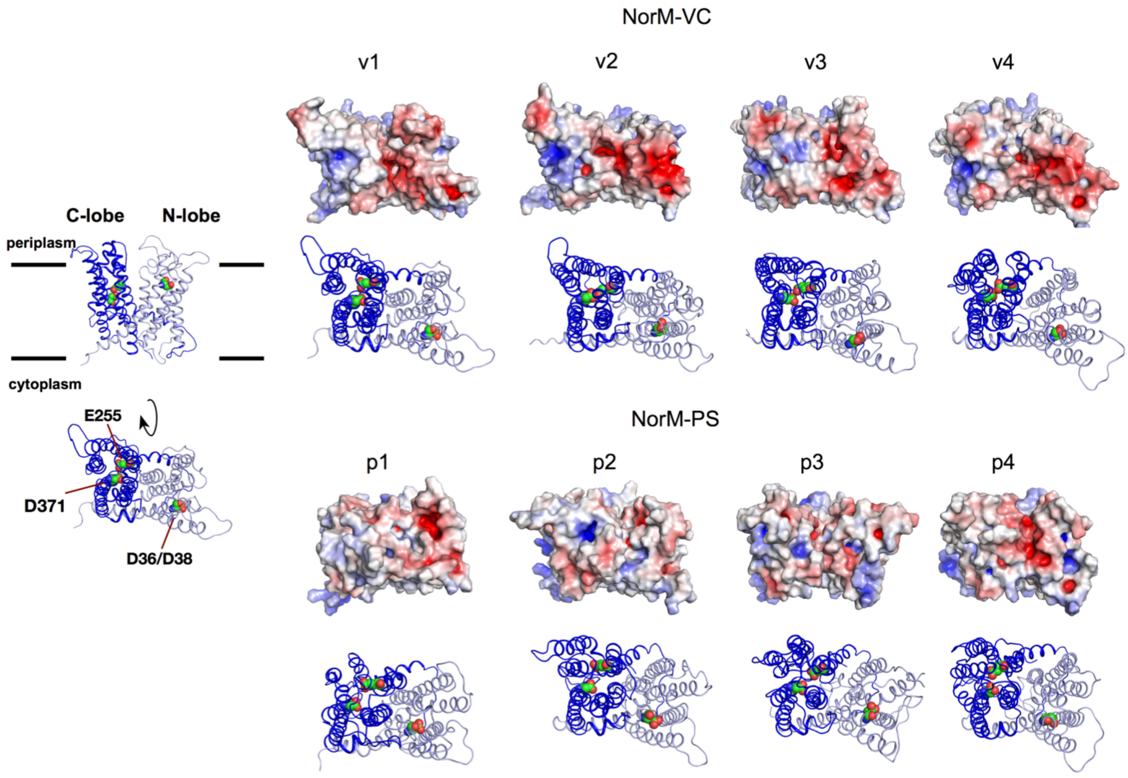

Figure S2. Comparison of the external surface of NorM-VC and NorM-PS. Electrostatic surface potentials of the protein surface at the periplasmic face of outward-facing NorM-VC (top row) and NorM-PS (bottom row) at the end of four 500 ns equilibrium simulations (v1 to v4 for NorM-VC and p1 to p4 for NorM-PS in Fig. S1). The molecular surfaces are coloured according to electrostatic potential from -5 (red) to +5 kT/e (blue). D36, E255 and D371 in NorM-VC and D38, E257 and D373 in NorM-PS are shown in sphere representation. Figure was generated using Pymol v2.4.0.

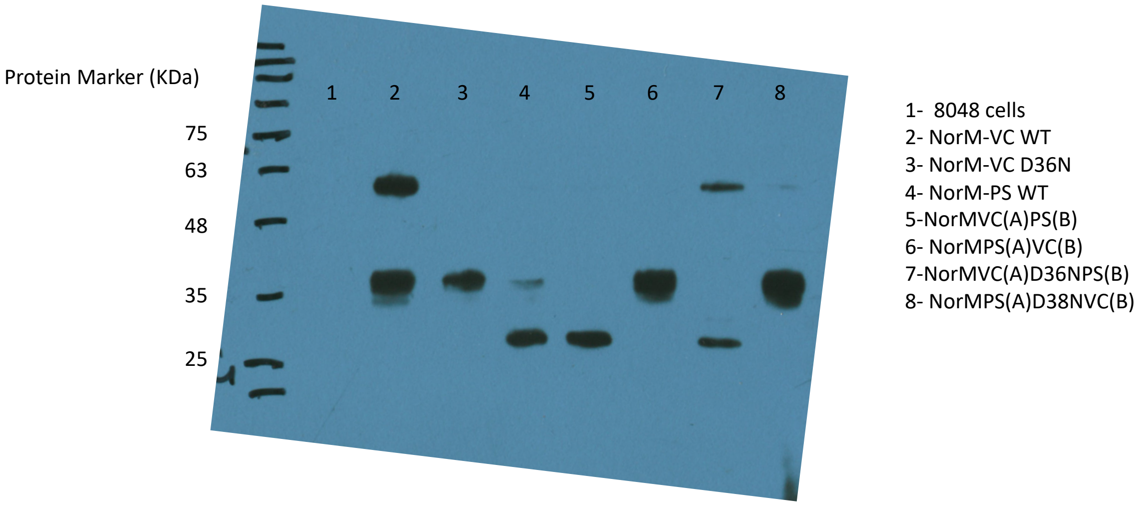

Figure S3. Uncropped immunoblot. Complete immunoblot of lactococcal membrane vesicles without expressed NorM proteins (lane 1) or containing wildtype NorM-VC (lane 2), NorM-VC-D36N (lane 3), NorM-PS (lane 4), *n*VC-*c*PS (lane 5), *n*PS-*c*VC (lane 6), *n*VC-*c*PS-D36N (lane 7) and *n*PS-*c*VC-D38N (lane 8), utilised in Fig. 8b.

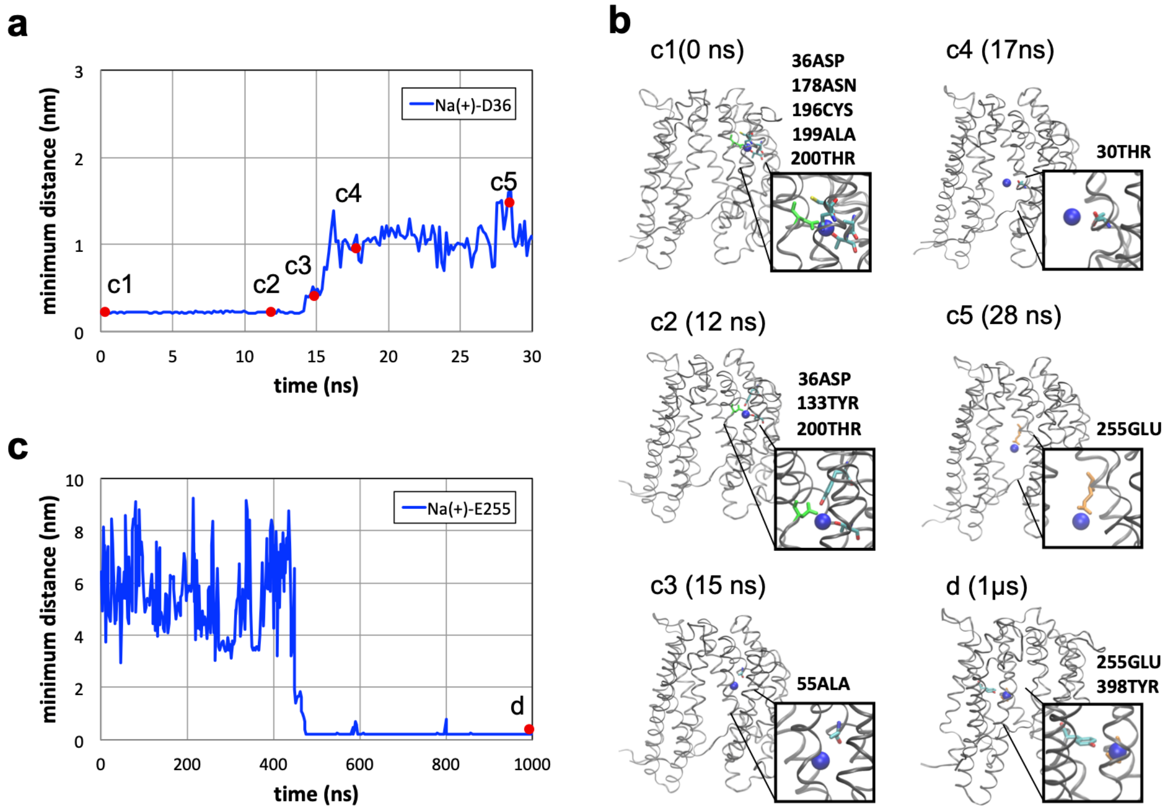

Figure S4. Na$^+$ binding to inward-facing NorM-VC. (**a**) Minimum distance between Na$^+$ and D36 (with D36, E255 and D371 deprotonated). Na$^+$ moves from D36 to E255 via a cavity region containing water molecules rather than residues in close vicinity. (**b**) Snapshots at 0 ns (c1) to 28 ns (c5) of Na$^+$ translocation from D36 to E255, and snapshot at 1 μs (d) of Na$^+$ binding to E255 from the cytoplasmic side (with protonated D371). The black letters indicate the residues (in stick representation) that are within 0.35 nm of the moving Na$^+$. (**c**) Minimum distance between Na$^+$ and E255 (with protonated D371). In the simulation, Na$^+$ moves from the cytoplasmic side and is bound near E255 until the end of the simulation (1 μs).

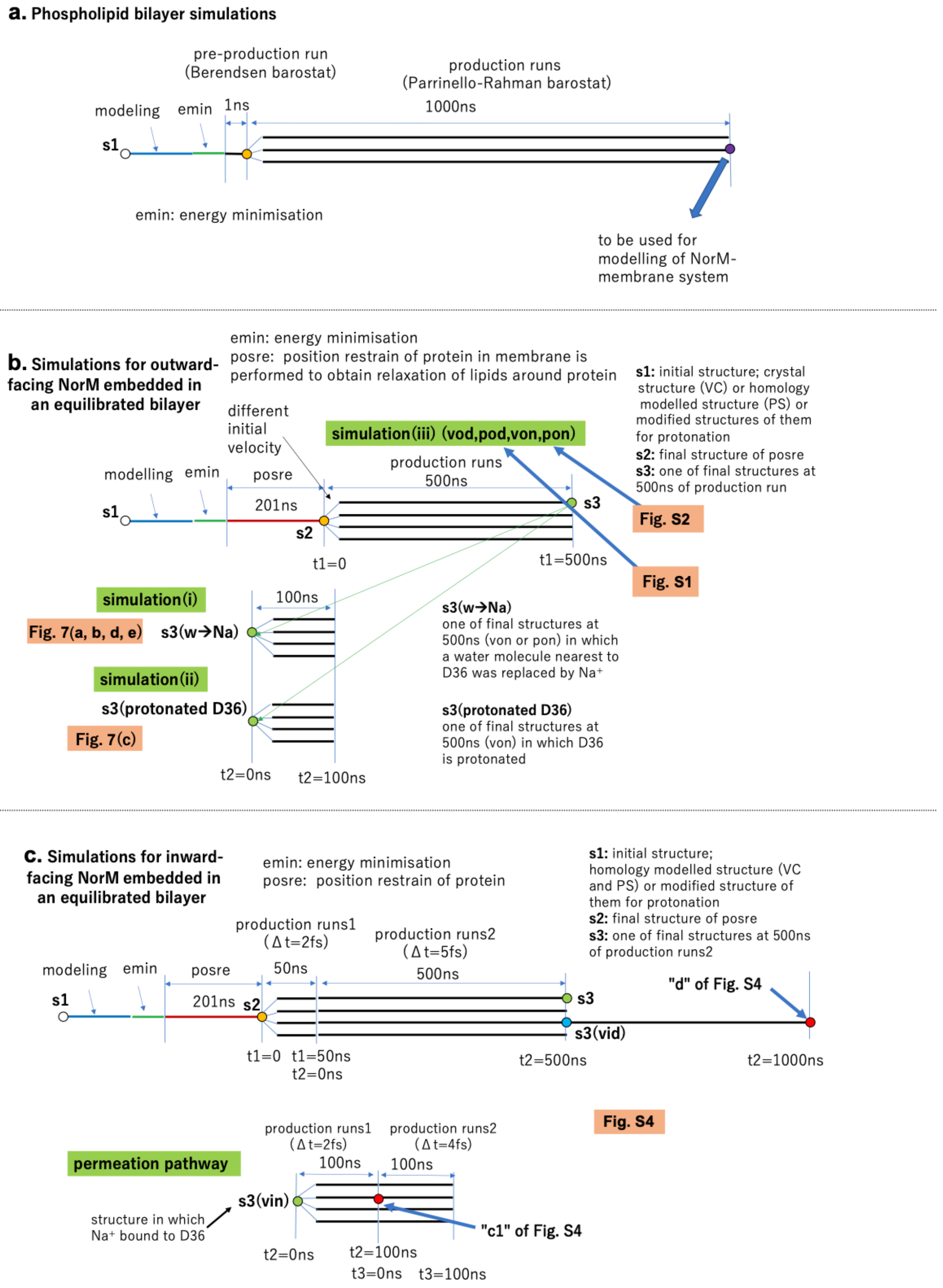

Figure S5. Schematic summarising the sequential steps in MD simulations in this study. See main text and Methods for detailed descriptions.
