# Supplementary material for: Engineered MATE multidrug transporters reveal two functionally distinct ion-coupling pathways in NorM from Vibrio cholerae
Source: Commun Biol. 2021 May 11;4:558. doi: 10.1038/s42003-021-02081-6 (PMC8113278; doi:10.1038/s42003-021-02081-6)
Supplement: Supplementary file 2 — Reporting Summary [file 42003_2021_2081_MOESM2_ESM.pdf]

nature research

Corresponding author(s): Dr Hendrik W. van Veen

Last updated by author(s): Mar 17, 2021

Reporting Summary

Nature Research wishes to improve the reproducibility of the work that we publish. This form provides structure for consistency and transparency in reporting. For further information on Nature Research policies, see our Editorial Policies and the Editorial Policy Checklist.

Statistics

For all statistical analyses, confirm that the following items are present in the figure legend, table legend, main text, or Methods section.

n/a	Confirmed	
☐	☒	The exact sample size (n) for each experimental group/condition, given as a discrete number and unit of measurement
☐	☒	A statement on whether measurements were taken from distinct samples or whether the same sample was measured repeatedly
☐	☒	The statistical test(s) used AND whether they are one- or two-sided<br>*Only common tests should be described solely by name; describe more complex techniques in the Methods section.*
☒	☐	A description of all covariates tested
☐	☒	A description of any assumptions or corrections, such as tests of normality and adjustment for multiple comparisons
☐	☒	A full description of the statistical parameters including central tendency (e.g. means) or other basic estimates (e.g. regression coefficient) AND variation (e.g. standard deviation) or associated estimates of uncertainty (e.g. confidence intervals)
☒	☐	For null hypothesis testing, the test statistic (e.g. F, t, r) with confidence intervals, effect sizes, degrees of freedom and P value noted<br>*Give P values as exact values whenever suitable.*
☒	☐	For Bayesian analysis, information on the choice of priors and Markov chain Monte Carlo settings
☒	☐	For hierarchical and complex designs, identification of the appropriate level for tests and full reporting of outcomes
☒	☐	Estimates of effect sizes (e.g. Cohen's d, Pearson's r), indicating how they were calculated

Our web collection on statistics for biologists contains articles on many of the points above.

Software and code

Policy information about availability of computer code

Data collection	Fluorescence traces were collected in FLWinlab v4.0 software and exported to MS Excel v16.42. MD simulations were conducted in GROMACS v2018.4.
Data analysis	Fluorimetry and biochemical data were analyzed in Graphpad Prism v8.4.3. Structural data were analyzed in Pymol v2.4.0. For MD simulations, data were analyzed using tools in GROMACS v2018.4 and Pymol v2.4.0. Sequence alignments were generated in Clustal Omega v1.2.4

For manuscripts utilizing custom algorithms or software that are central to the research but not yet described in published literature, software must be made available to editors and reviewers. We strongly encourage code deposition in a community repository (e.g. GitHub). See the Nature Research guidelines for submitting code & software for further information.

Data

Policy information about availability of data

All manuscripts must include a data availability statement. This statement should provide the following information, where applicable:

- Accession codes, unique identifiers, or web links for publicly available datasets
- A list of figures that have associated raw data
- A description of any restrictions on data availability

MD simulations were based on the atomic coordinates of NorM-VC (PDB-ID: 3MKT). The datasets generated during and/or analysed during the current study have been deposited in the University of Cambridge research repository Apollo with DOI link https://doi.org/10.17863/CAM.64600 or are available from the corresponding author upon reasonable request.

Field-specific reporting

Please select the one below that is the best fit for your research. If you are not sure, read the appropriate sections before making your selection.

☒ Life sciences ☐ Behavioural & social sciences ☐ Ecological, evolutionary & environmental sciences

For a reference copy of the document with all sections, see nature.com/documents/nr-reporting-summary-flat.pdf

Life sciences study design

All studies must disclose on these points even when the disclosure is negative.

Sample size	All experiments were repeated 3 times or more as indicated in the figure legends and the Materials and Methods section on Statistics and reproducibility.
Data exclusions	No data were excluded.
Replication	Reproducibility of data was tested by using independent batches of cells and protein samples, which were collected and prepared on independent days.
Randomization	This is a biochemical study using populations of cells and purified proteins. No experiments focussed on individual cells or proteins.
Blinding	As group allocations were not part of this study, blinding was not relevant.

Reporting for specific materials, systems and methods

We require information from authors about some types of materials, experimental systems and methods used in many studies. Here, indicate whether each material, system or method listed is relevant to your study. If you are not sure if a list item applies to your research, read the appropriate section before selecting a response.

Materials & experimental systems

n/a	Involved in the study	
☐	☒	Antibodies
☒	☐	Eukaryotic cell lines
☒	☐	Palaeontology and archaeology
☒	☐	Animals and other organisms
☒	☐	Human research participants
☒	☐	Clinical data
☒	☐	Dual use research of concern

Methods

n/a	Involved in the study	
☒	☐	ChIP-seq
☒	☐	Flow cytometry
☒	☐	MRI-based neuroimaging

Antibodies

Antibodies used	Western blots were performed using mouse anti-polyhistidine tag antibody (Sigma-Aldrich, cat. no.: H1029) and goat antimouse antibody (Sigma-Aldrich, cat. no.: A4416).
Validation	These antibodies are very frequently used by ourselves and many others in the field.
